# Knockdown of RRM1 in tumor cells promotes radio-/chemotherapy induced ferroptosis by regulating p53 ubiquitination and p21-GPX4 signaling axis

**DOI:** 10.1038/s41420-022-01140-z

**Published:** 2022-08-01

**Authors:** Yang Gao, Bin Chen, Ruru Wang, An Xu, Lijun Wu, Huayi Lu, Guoping Zhao

**Affiliations:** 1 High Magnetic Field Laboratory, Key Laboratory of High Magnetic Field and Ion Beam Physical Biology, Chinese Academy of Sciences; Anhui Province Key Laboratory of Environmental Toxicology and Pollution Control Technology, Hefei Institutes of Physical Science, Chinese Academy of Sciences, Hefei, Anhui China; 2 Guangdong Provincial Key Laboratory of Infectious Diseases and Molecular Immunopathology, Shantou University Medical College, Shantou, Guangdong China; 3 Information Materials and Intelligent Sensing Laboratory of Anhui Province, Institutes of Physical Science and Information Technology, Anhui University, Hefei, Anhui China; 4 The First Affiliated Hospital of USTC, Division of Life Sciences and Medicine, University of Science and Technology of China, Hefei, Anhui China

**Keywords:** Radiotherapy, Predictive markers, Cell death, Cancer therapeutic resistance

## Abstract

Ferroptosis, a type of regulated cell death brought about by lipid peroxidation, has been discovered to suppress tumor growth. Here, we report that targeting RRM1 promotes ferroptosis and affects sensitivity to radiation and chemotherapeutics in cancer cells. In vitro experiments demonstrate that RRM1 increases the accumulation of cellular reactive oxygen species (ROS) and lipid peroxidation by disrupting the activity and expression of the antioxidant enzyme GPX4. Further studies reveal the downstream mechanisms of RRM1, which can regulate the deubiquitinating enzyme USP11 and ubiquitinating enzyme MDM2 to affect the ubiquitination modification of p53. Unstable p53 then inhibited the activity and expression of GPX4 by restraining the p21 protein. Furthermore, our data reveal that targeting RRM1 also increases radiation-induced DNA damage and apoptotic signaling and causes crosstalk between ferroptosis and apoptosis. On the basis of our collective findings, we propose that RRM1 is an essential negative mediator of radiosensitivity through regulating ferroptosis, which could serve as a potential target to inhibit the tumor’s antioxidant system and enhance the efficiency of radio/chemotherapy.

## Introduction

Radiation resistance has yet to be fully elucidated. Cancer cells accumulate resistance to radiation by activating intrinsic mechanisms, such as DNA repair and apoptosis inhibition [1, 2]. Radiation-induced reactive oxygen species (ROS) damage nucleic acids, proteins, and lipids in many ways [3, 4]. However, tumor cells can acquire the ability to adapt to ROS by synthesizing more antioxidants [5, 6]. Therefore, targeting cellular antioxidant defense systems enhances radiosensitivity [7].

Ferroptosis is caused by ROS and lipid peroxidation, which differs from apoptosis in its morphology and mechanism [8, 9]. As a cellular process, ferroptosis is an essential factor for both normal development and various diseases, including cancer [10, 11]. As a central regulator of ferroptosis, glutathione peroxidase 4 (GPX4) converts lipid hydroperoxides into lipid alcohols using reduced glutathione, thereby reducing lipid peroxidation and preventing ferroptosis [12, 13]. Current studies show that the production of ROS and the expression of the lipid metabolism enzyme ACSL4 are the main mechanisms through which radiation induces ferroptosis in cancer cells [14]. Notably, the radiation also induces the expression of GPX4, which likely represents a potential adaptive response and may contribute to radioresistance.

Using our previously established dbCRSR database (dbCRSR, http://www.xialab.info:8080/dbCRSR/) [15], after constructing the functional network by integrating protein-protein interaction (PPI) and gene co-expression information, a radiosensitivity gene RRM1 (Ribonucleotide reductase subunit M1) was identified. As a key enzyme catalyzing the transformation of ribonucleotide diphosphates to deoxyribonucleoside diphosphates, RRM1 is involved in cell migration, tumor and metastasis development, and DNA synthesis [16, 17]. Studies have shown that RRM1 is recruited to DSB sites to ensure a balanced supply of dNTPs during mammalian DNA repair [18]. Gautam et al. found that RRM1 remains relatively constant throughout the cell cycle, suggesting RRM1 may have functions outside the formation of ribonucleotide reductases [19]. Recently, it has been reported that UVA-induced ROS can transiently and reversibly oxidize the RRM1 subunit, which may depend on singlet oxygen production and the presence of intracellular GSH [20]. Therefore, it is critical to study how RRM1 maintains the balance between antioxidants and ROS for radiation-induced ferroptotic cell death.

As a critical tumor suppressor, p53 activates multiple target genes and coordinates numerous reactions to suppress tumorigenesis, such as arresting the cell cycle, causing senescence, and inducing apoptosis [21]. A recent function ascribed to p53 is the regulation of ferroptosis, and it has a dual role in controlling ferroptosis [22]. It has been reported that p53 induces ferroptosis by inhibiting SLC7A11 transcription [23]. In contrast, activating the p53-p21 pathway makes cells more resistant to ferroptosis [24], but the specific mechanism is unclear.

In this study, we identified that RRM1 regulates radiation and chemotherapeutics induced ferroptosis. RRM1 deficiency impairs the stability of p53 and sensitizes different types of cancer cells to ferroptosis by reducing GPX4 expression. Mechanistically, knockdown of RRM1 stimulates the binding of the ubiquitinating enzyme MDM2 and p53 while inhibiting the binding of the deubiquitinating enzyme USP11 to p53, thereby increasing the ubiquitination of p53. The instability of p53 results in lower expression of p21, which causes an increase in lipid ROS and a decrease in cell survival time by inhibiting GPX4. Additionally, we verified that targeting RRM1 could also increase radiation-induced apoptosis signals and cause crosstalk between radiation-induced ferroptosis and apoptosis.

## Results

### RRM1 is highly expressed upon radiation

**Fig. 1:**
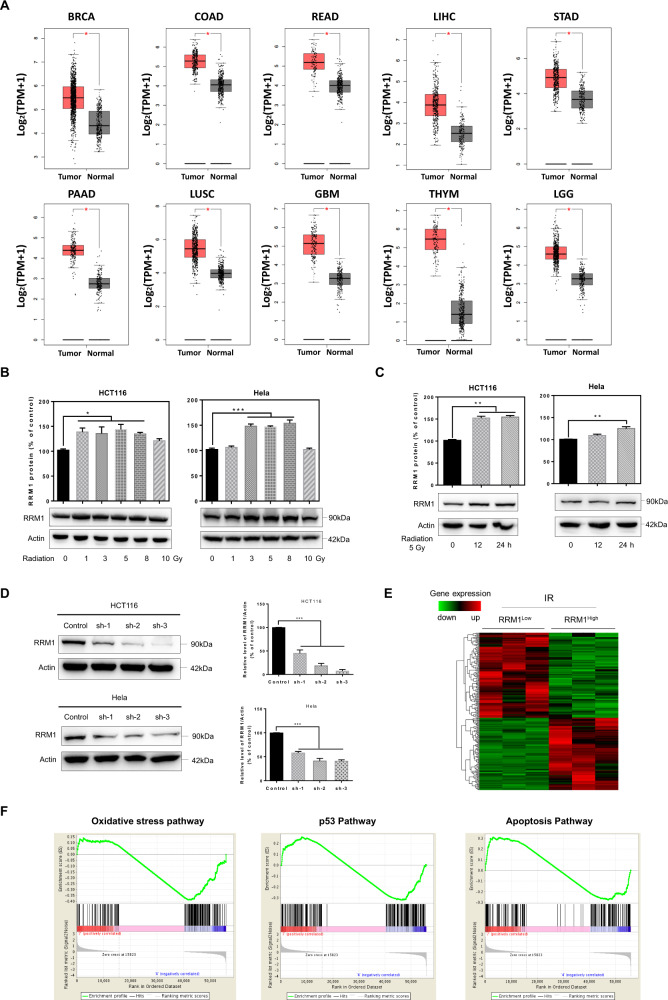
RRM1 is highly expressed after radiation. **A** TCGA data show that RRM1 is overexpressed in multiple cancer types. **B**, **C** Cells were irradiated with γ-ray at different doses (**B**) or 5 Gy (**C**), and the protein levels of RRM1 were detected after 24 h (**B**) or at different time points (**C**). **D** RRM1 expression was stably reduced by lentiviral infection. **E** Heatmap from the 224 differentially expressed genes comparing two groups: RRM1^Low^ to RRM1^High^. **F** Gene Set Enrichment Assay (GSEA) compares two phenotypes, RRM1^Low^ and RRM1^High^.

Based on The Cancer Genome Atlas (TCGA) enriched dataset, we analyzed the expression levels of RRM1 in human cancers. It was shown that the RRM1 expression was higher in the different clinical samples than in the corresponding normal tissues (Fig. 1A). Next, we examined the effect of γ-ray on RRM1 expression in cancer cells. With the increase of radiation dose, the expression level of RRM1 increased significantly (Fig. 1B). After a fixed-dose (5 Gy), RRM1 expression also increased over time (Fig. 1C). Additionally, although RRM1 is less expressed in non-cancerous cells (HIEC cells), it is still up-regulated by radiation (Fig. S1A, B). Overall, RRM1 was overexpressed in cancer tissues compared with normal tissues and was up-regulated upon radiation.

To identify genes or pathways associated with RRM1 after radiation, we conducted RNA sequencing analysis. Short-hairpin RNA (shRNA) was used to knockdown RRM1 in HCT116 and Hela cells (Fig. 1D). After IR treatment, 224 genes were differentially expressed in RRM1^Low^ cells compared to RRM1^High^ cells (Fig. 1E). Furthermore, Gene Set Enrichment Analysis (GSEA) compared all genes between RRM1^Low^ group and RRM1^High^ group (Fig. 1F). The RRM1^low^ phenotype is significantly enriched for gene sets associated with oxidative stress pathway, p53 pathway, and apoptosis pathway.

### Target RRM1 promotes radiation-induced ferroptosis

**Fig. 2:**
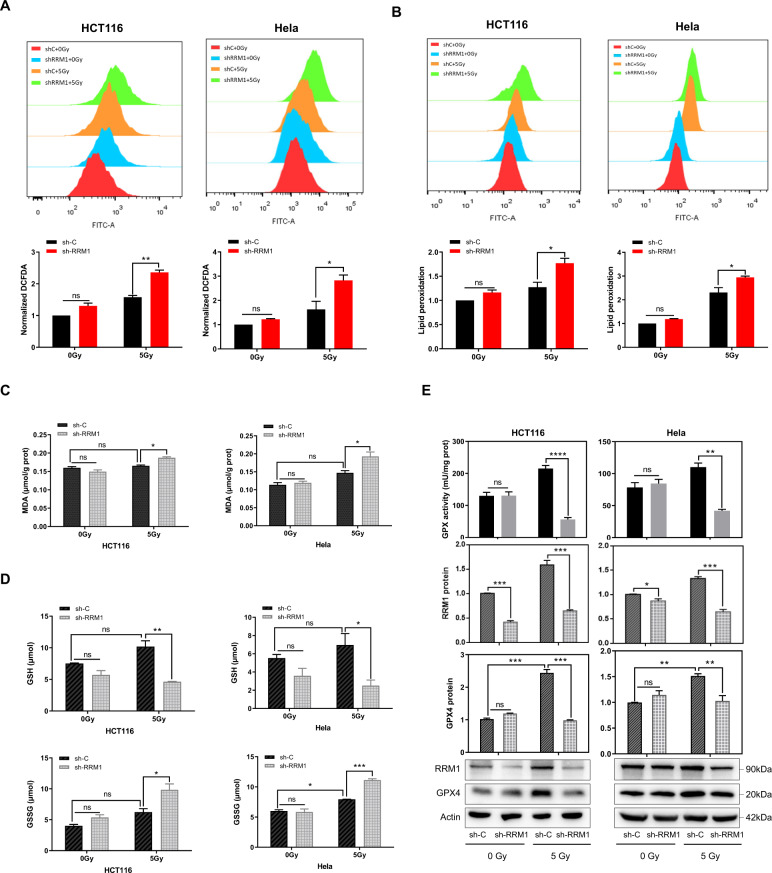
Target RRM1 promotes IR-induced ferroptosis. **A**, **B** Assessment of total ROS levels (**A**: DCFDA staining) or lipid peroxidation (**B**: C11-BODIPY staining) in RRM1 knockdown cells after radiation. **C** Knockdown of RRM1 increased radiation-induced MDA production. **D** The levels of GSH and GSSG were assayed in RRM1 knockdown cells 24 h after 5 Gy γ-ray. **E** Lower GPX activity and GPX4 expression were measured in RRM1 knockdown cells 24 h after 5 Gy γ-ray.

Radiation induces ferroptosis in cancer cells, and both radiation and ferroptosis are associated with ROS [14]. To verify whether RRM1 regulates radiation-induced ferroptosis, we performed a series of experiments. As shown in Fig. 2A, the knockdown of RRM1 increased the ROS levels induced by radiation in cancer cells. Ferroptosis is characterized by lipid peroxidation [8]. Indeed, we found that RRM1 knockdown cells increased radiation-induced lipid peroxidation and MDA (Malondialdehyde) levels (Fig. 2B, C). Furthermore, ferroptotic events such as glutathione (GSH) depletion and glutathione disulfide (GSSG) generation are promoted (Fig. 2D). Moreover, GPX4, a glutathione peroxidase that can inhibit ferroptosis, was highly expressed after radiation (Fig. 2E). However, compared with control shRNA cells, GPX4 was significantly decreased in RRM1 knockdown cells upon radiation (Fig. 2E). In addition, the activity of GPX was markedly inhibited in knockdown RRM1 cells after radiation (Fig. 2E). Taken together, those above results show that targeting RRM1 promotes radiation-induced ferroptosis.

### RRM1 regulates the ubiquitination of p53

**Fig. 3:**
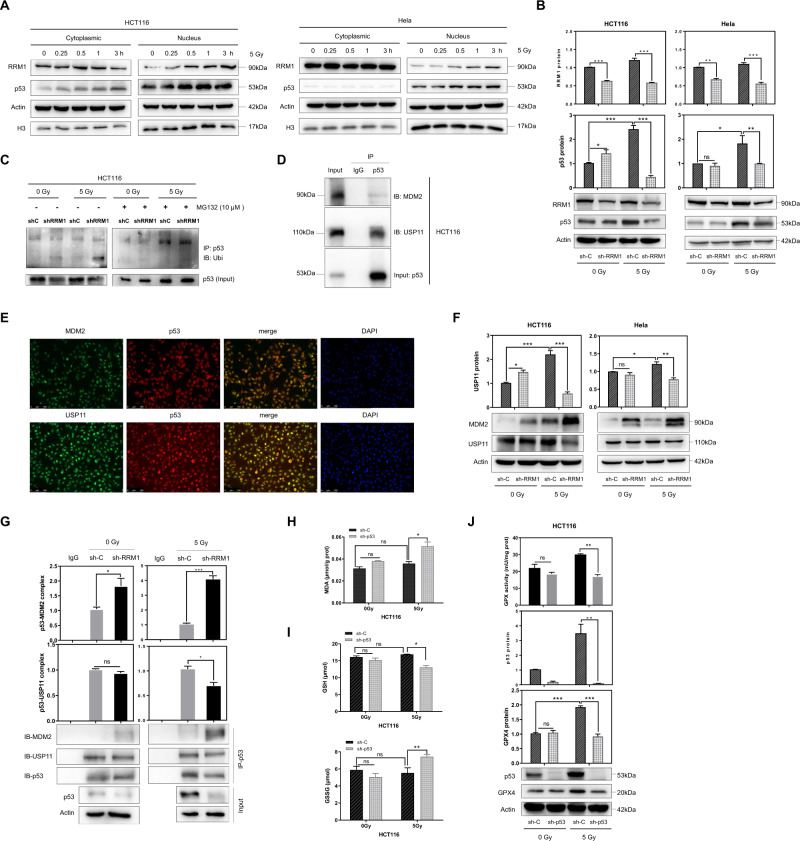
RRM1 regulates the ubiquitination of p53. **A** Cells were treated with 5 Gy γ-ray for nucleocytoplasmic separation at different time points. **B** The expression level of p53 was detected in RRM1 knockdown cells after 5 Gy γ-ray. **C** Cells were treated with MG132, p53 was immunoprecipitated with anti-p53 antibody and immunoblotted with anti-ubiquitin antibody. **D**, **E** Co-IP (**D**) or immunofluorescence (**E**) were used to detect the interaction (**D**) or co-localization (E) of endogenous p53 and endogenous MDM2 or USP11 in HCT116 cells (**D**) or Hela cells (**E**). **F** Expression levels of MDM2 and USP11 proteins in RRM1 knockdown cells after 5 Gy γ-ray. **G** Analysis of the binding between p53 and MDM2 or USP11 in control and RRM1 knockdown cells by immunoprecipitation and immunoblotting. **H**, **I** MDA (**H**) and GSH and GSSG levels (**I**) were detected in p53-deficient cells after radiation. **J** Lower GPX activity and GPX4 expression were detected in RRM1 knockdown cells after 5 Gy γ-ray.

Our data provide strong support that RRM1 regulated the radiosensitivity of cancer cells by triggering ferroptosis. However, the potential mechanism by which RRM1 regulates this process remains unclear. We found that radiation triggered the nuclear aggregation of RRM1, which was initially located in the cytoplasm (Fig. 3A). In addition, after radiation, p53 also accumulates in the nucleus (Fig. 3A). Next, the expression level of p53 protein was examined to elucidate the relationship between RRM1 and p53 in radiation-induced ferroptosis. In HCT116 and Hela cells, p53 expression was significantly increased after radiation, but RRM1 knockdown inhibited this effect (Fig. 3B). RRM1-mediated the decreasing of p53 stability reduced the expression level of p53 protein. As expected, knockdown of RRM1 was shown to promote p53 polyubiquitination after radiation (Fig. 3C).

The stability of p53 protein is precisely regulated by the ubiquitin-proteasome system. As a negative regulator of p53, MDM2 ubiquitinates and degrades p53 [25]. Ubiquitin-specific protease USP11 deubiquitinates and stabilizes p53 [26]. The interaction of p53 with MDM2 or USP11 was confirmed (Fig. 3D, E). The expression of MDM2 was significantly elevated in RRM1 knockdown cells upon radiation, while the expression of USP11 was decreased. Furthermore, we evaluated the interaction between p53 and USP11 or MDM2 in control and RRM1 knockdown cells, respectively. As shown in Fig. 3G, knockdown of RRM1 enhanced the p53/MDM2 interaction and weakened the p53/USP11 interaction after radiation treatment. Together, these data indicate that RRM1 regulates p53 ubiquitination through MDM2 and USP11.

In addition, higher MDA levels were detected in p53-deficient cells after radiation (Fig. 3H). Meanwhile, the depletion of GSH, the production of GSSG, the activity of GPX, and the expression of GPX4 all indicate that p53 deficiency promotes radiation-induced ferroptosis (Fig. 3I, J).

### RRM1 regulates GPX4 by inhibiting the p53-p21 signal axis

**Fig. 4:**
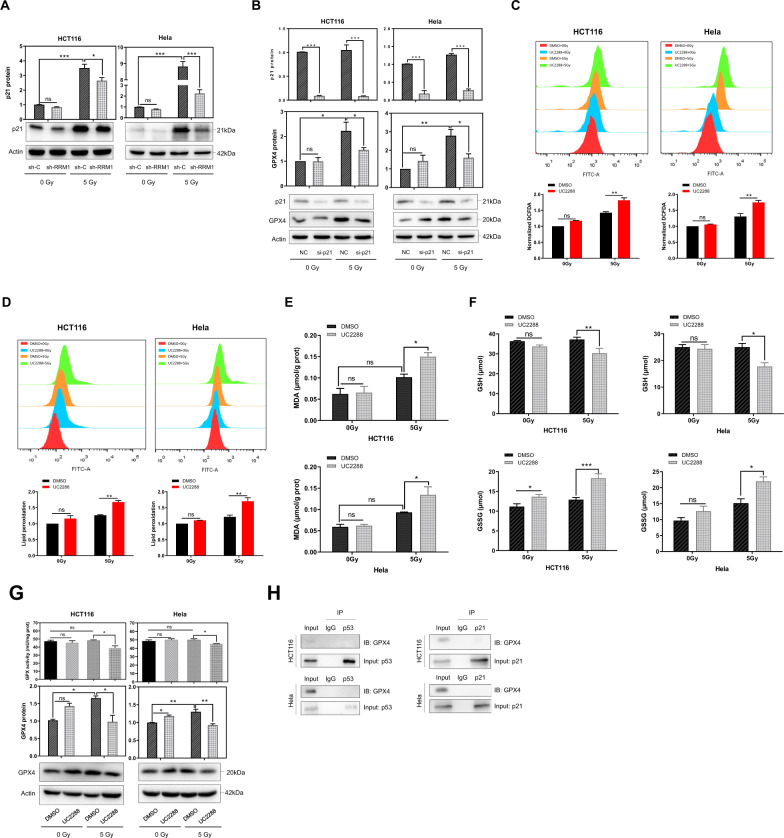
RRM1 regulates GPX4 by inhibiting the p53-p21 signal axis. **A** p21 protein was detected in RRM1 knockdown cells after exposure to 5 Gy γ-ray. **B** Low expression of GPX4 protein was analyzed in p21 knockdown cells after 5 Gy γ-ray. **C**–**F** Total ROS levels (**C**), lipid peroxidation (**D**), MDA (**E**), and GSH and GSSG levels (**F**) were detected in HCT116 and Hela cells at 24 h after exposure to 1 µM UC2288 and 5 Gy γ-ray. **G** Lower GPX activity and GPX4 expression were detected in control and 5 µM UC2288 treated cells upon 5 Gy γ-ray. **H** The interaction of endogenous GPX4 with p53 or p21 was examined by Co-IP assay.

As a canonical target gene of p53, p21 (also known as CDKN1A) is involved in several cellular activities, such as cellular stress responses, metabolism, and cell cycle [27]. We found that RRM1 knockdown combined with radiation decreased p21 protein levels (Fig. 4A). The p53-p21 pathway can promote cancer cell survival following serine deprivation by increasing glutathione levels and maintaining redox balance [24, 28]. To examine the role of p21 in radiation-induced ferroptosis, siRNA and the inhibitor UC2288 were used, respectively. Both of them can successfully reduce p21 expression (Fig. S2A, B, Supplementary Materials). As shown in Fig. 4B, the expression levels of GPX4 were decreased in p21 knockdown cells compared with control cells upon radiation. Similarly, the p21 inhibitor UC2288 increased radiation-induced oxidative stress and lipid peroxidation (Fig. 4C, D) and markedly promoted a series of radiation-induced ferroptosis events, including MDA production (Fig. 4E), GSH consumption, and GSSG generation (Fig. 4F), reduced GPX activity, and inhibition of GPX4 expression (Fig. 4G). Furthermore, compared with non-irradiated cells, the p21 agonist LAQ824 increased GPX4 expression after radiation (Fig. S2C, D, Supplementary Materials). In addition, we demonstrated that GPX4 did not interact with p53 or p21 protein in HCT116 and Hela cells using Co-IP methods (Fig. 4H). Collectively, our results indicate that inhibition of the p53-p21 signaling axis indirectly inactivates GPX4 through GSH depletion.

### RRM1 regulates chemosensitivity in cancer cells

**Fig. 5:**
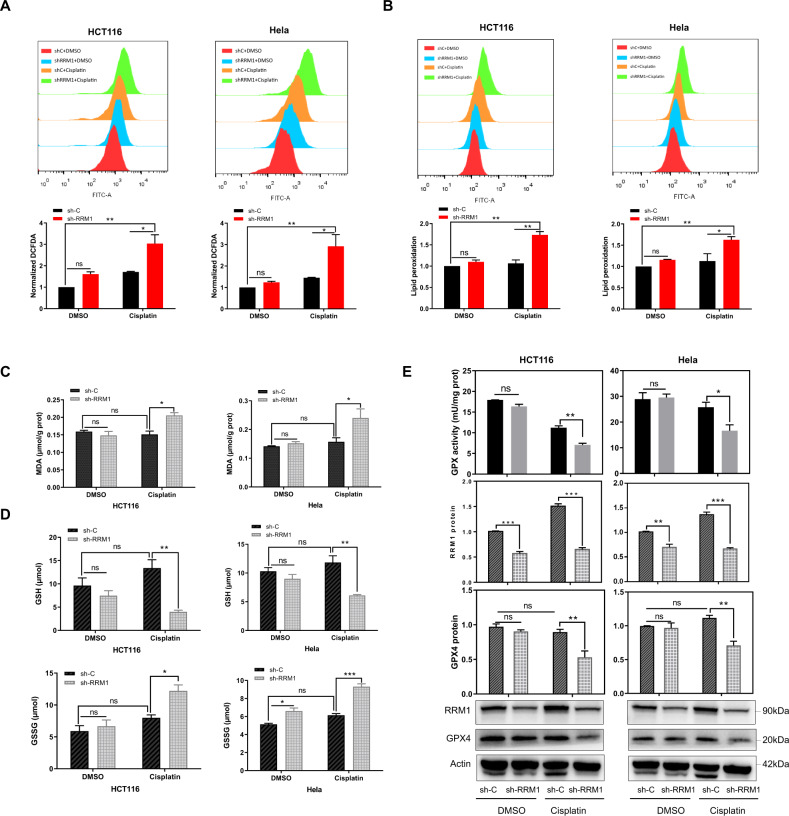
RRM1 mediates chemosensitivity in cancer cells. **A**, **B** Total ROS (A) and lipid peroxidation (B) were measured in RRM1 knockdown cells at 48 h after exposure to 5 µg/mL cisplatin. **C** Knockdown of RRM1 increased cisplatin-induced MDA production. **D** The levels of GSH and GSSG were assayed in RRM1 knockdown cells after treatment with 5 µg/mL cisplatin. **E** Lower GPX activity and GPX4 expression were detected in RRM1 knockdown cells 48 hours after 5 µg/mL cisplatin treatment.

Cisplatin is an extremely effective and widely used anti-cancer drug. However, many cancers have developed resistance to cisplatin therapy [29, 30]. Cisplatin can induce ferroptosis in A549 and HCT116 cells at appropriate concentrations [31]. To determine whether RRM1 also regulates cisplatin-induced ferroptosis, we performed combination treatment studies with RRM1 knockdown and cisplatin. As predicted by our hypothesis, RRM1 knockdown increased the production of oxidative stress and lipid peroxidation in cisplatin-induced cells (Fig. 5A, B). Our results also demonstrated that suppression of RRM1 significantly promoted cisplatin-induced ferroptotic events, including MDA production, GSH depletion, and GSSG generation (Fig. 5C, D). Moreover, the expression of GPX4 and the activity of GPX were significantly reduced after cisplatin treatment combined with RRM1 knockdown (Fig. 5E). In addition, it has been reported that gemcitabine is a chemotherapeutic drug targeting RRM1 [32]. Combined gemcitabine with ferroptosis inducer erastin increased oxidative stress and lipid peroxidation of cancer cells and decreased the expression level of SLC7A11 protein (Fig. S3, Supplementary Materials). Taken together, these data indicate that RRM1 inhibition combined with cisplatin can promote the occurrence of ferroptosis, thereby regulating the chemosensitivity in cancer cells.

### RRM1 regulates radiation-induced apoptotic signaling and causes crosstalk between ferroptosis and apoptosis

**Fig. 6:**
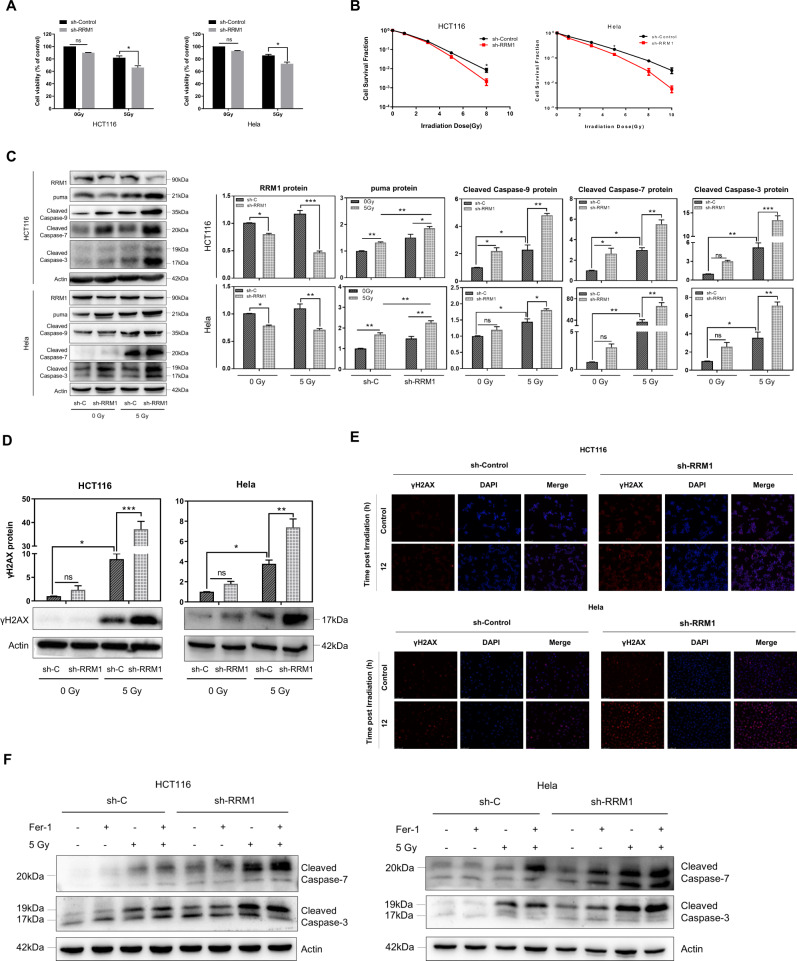
RRM1 regulates the radiation-induced endogenous apoptosis pathway. **A**, **B** Cell viability (**A**) and colony formation (**B**) were examined in RRM1 knockdown cells after 5 Gy γ-ray. **C** After radiation, the expression levels of puma and apoptotic proteins (cleaved caspase-9/7/3) were detected in RRM1 knockdown cells. **D**, **E** γH2AX protein was analyzed by western blot (**D**) and immunofluorescence staining (**E**) after radiation. **F** Expression of apoptotic protein (cleaved caspase-3/7) after radiation combined with ferrostatin-1 (5 µM).

We found that RRM1 was overexpressed in the tumor. Furthermore, gene set enrichment analysis revealed that the apoptotic pathway was enriched in RRM1 knockdown cells (Fig. 1F). Therefore, we investigated the apoptotic signaling of radiation in RRM1 knockdown cells. As shown in Fig. 6A, B, cell viability and colony formation in cancer cells were reduced when the radiation was combined with RRM1 knockdown. Consistently, high levels of apoptotic protein expression (cleaved caspase-3, cleaved caspase-7, and cleaved caspase-9) were detected in RRM1-knockdown cells after radiation (Fig. 6C). Notably, the expression of puma protein was also significantly increased (Fig. 6C). Furthermore, RRM1 knockdown significantly promoted the expression of γH2AX protein, a marker of DNA damage upon radiation (Fig. 6D, E). The above results indicate that the downregulation of RRM1 promotes radiation-induced apoptotic signaling.

Ferroptosis is thought to be distinct from cell death types such as necrosis, autophagy, apoptosis, necroptosis, but recent studies have revealed the interplay between ferroptosis and apoptosis [33]. To confirm the relationship between radiation-induced ferroptosis and apoptosis mediated by RRM1, HCT116 cells and Hela cells were treated with the ferroptosis inhibitor ferrostatin-1 and activated cysteine proteases were tested. Compared with radiation alone, a higher expression of apoptotic proteins (cleaved caspase-3, cleaved caspase-7) was detected in RRM1-knockdown cells after radiation with ferrostatin-1 treatment (Fig. 6F). Notably, in control vector cells, highly expressed apoptotic proteins were also detected upon radiation and ferrostatin-1 treatment. The above results indicate that RRM1 causes the crosstalk of radiation-induced apoptosis and ferroptosis. That is, when inhibiting the radiation-induced ferroptosis, the radiation-induced apoptosis signal compensatory increased.

## Discussion

RRM1 has been less studied recently, even though it is a critical factor. Therefore, apart from providing dNTPs for DNA synthesis, the biological function of RRM1 is still unknown [18]. Recent data indicate that UVA-induced ROS can transiently and reversibly oxidize the RRM1 subunit, suggesting that RRM1 may be involved in oxidative stress [20]. Here, we demonstrate for the first time a direct connection between RRM1 and radio-/chemotherapy-induced ferroptosis in cancer cells. Our study identified RRM1 as a negative regulator of radio-/chemotherapy-induced ferroptosis. RRM1 increases the instability of p53 by regulating the physical interaction of p53 with the ubiquitinating enzyme MDM2 and the deubiquitinating enzyme USP11, subsequently suppressing p21 and GPX4, thereby promoting the accumulation of lipid peroxidation and occurrence of radiation-induced ferroptosis. In summary, our findings offer new mechanistic insight into the regulation of RRM1 and indicate that the lack of RRM1 exacerbates ferroptosis of cancer cells upon DNA damage, and targeting RRM1 improves radio-/chemotherapy.

Accumulating evidence has demonstrated that tumor cells are prone to synthesize more antioxidants to resist radiation-induced ROS [5, 6]. Our findings show that radiation significantly increases the expression and activity of the antioxidant enzyme GPX4, which is consistent with previous studies [14]. This adaptive response of GPX4 may promote the survival of cancer cells during radiotherapy, leading to radioresistance. Using the online GEPIA database analysis, we showed that high protein levels of RRM1 were observed in multiple types of human cancers. RNA sequencing and GSEA analysis indicate that the high expression of RRM1 after radiation may be associated with oxidative stress, DNA repair, apoptosis. Importantly, lower GPX4 expression and activity were detected in RRM1 knockdown cells after radiation, suggesting that the balance between ROS and the antioxidant system was disrupted, further supporting the role of RRM1 in targeting the tumor antioxidant system to increase radiosensitivity.

Our study revealed that p53 is ubiquitinated in RRM1 knockdown cells upon radiation. Subsequently, we determined that MDM2 and USP11 were up-regulated and down-regulated in RRM1 knockdown cells, respectively, after radiation. MDM2 and USP11 play a vital role in the process of p53 ubiquitination modification. As a negative regulator of p53, MDM2 ubiquitinates and degrades p53 [25]. Furthermore, the stability of p53 is also regulated by deubiquitinating enzymes (DUBs) [34]. As a positive regulator of p53, ubiquitin-specific protease USP11 can deubiquitinate and stabilize p53 [26]. Our data clearly show that a deficiency in RRM1 promotes the interaction between MDM2 and p53 and weak the binding of USP11 and p53, indicating that RRM1 regulates the stability of p53 through ubiquitination and de-ubiquitination system. Taken together, our findings strongly imply that RRM1 is a regulator of p53 stability after radiation.

As the “guardian of the genome,” p53 controls cell survival and division under various stresses [35]. Beyond regulating intracellular processes such as the cell cycle, autophagy, and apoptosis, recent studies have shown that p53 modulates ferroptosis through transcriptional or posttranslational mechanisms [23]. Studies have shown that p53 enhances ferroptosis by inhibiting SLC7A11 expression or promoting the expression of GLS2 and SAT1. Conversely, p53 can inhibit ferroptosis by inducing CDKN1A/p21 expression or directly attenuating DPP4 activity [22]. The dual role of p53 in controlling ferroptosis makes the molecular mechanisms of ferroptosis more complex than previously thought. In our study, compared with wild-type cells, p53-deficient cells promoted radiation-induced ferroptosis. These results further confirmed that the ferroptosis signal mediated by RRM1 is closely related to the ubiquitination degradation of p53.

It has been reported that increased expression of wild-type p53 reduced ferroptosis signaling under cystine deprivation, and transactivation of the canonical p53 target gene p21 may be involved in this process [24, 28]. We found that p21 acts an essential role in the radiation-induced ferroptosis regulated by p53. Inhibition of p21 promoted radiation-induced lipid peroxidation and ROS and triggered a series of ferroptotic events. Notably, neither p53 nor p21 interacted with GPX4. The above results indicate that the p53-p21 signaling axis increases radiation-induced ferroptosis through regulating the consumption of GSH and indirectly inactivating the antioxidant enzyme GPX4 in RRM1 knockdown cells. In addition, compared with control shRNA cells, the DNA damage signal and downstream apoptotic cascade were markedly increased in RRM1 knockdown cells upon radiation. Moreover, RRM1-mediated crosstalk between radiation-induced apoptosis and ferroptosis preliminarily suggested that there might be a reciprocal transformation network between the two forms of death.

**Fig. 7:**
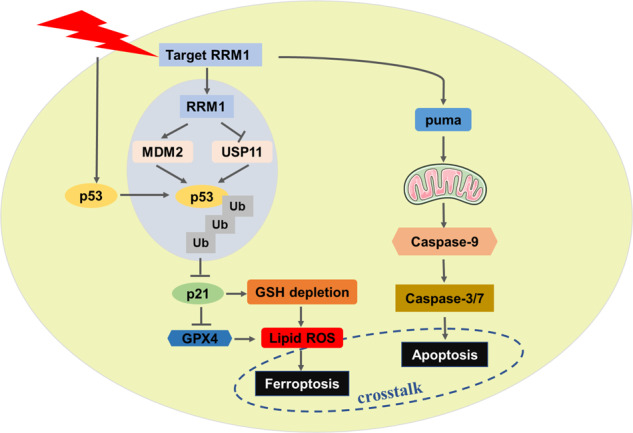
Schematic illustration of the proposed model. The mechanism of RRM1-mediated radiosensitization of cancer cells.

In summary, our study identified a novel regulatory role of RRM1, causing an imbalance of the antioxidant system in cancer cells to reverse radio- and chemoresistance (Fig. 7). The new function of RRM1 as a negative regulator of radio-/chemotherapy-induced ferroptosis can be used to design new cancer treatments. Future studies will explore the crosstalk mode between RRM1’s regulation of apoptosis and ferroptosis.

## Materials and methods

### Cell culture and radiation

HCT116, Hela, HEK293T, and HIEC cells were purchased from the ATCC. All cells were cultured in DMEM medium supplemented with 10% fetal bovine serum and 1% penicillin/streptomycin. We used a Biobeam GM gamma irradiator (Leipzig, Germany). The instrument is routinely maintained (dose rate = 3.27 Gy/min).

### ROS and lipid peroxidation analysis

Measurements were performed as previously described [14]. Cells were treated with 10 μM CM-H2DCFDA (ThermoFisher, C6827) (to detect ROS) or 5 μM BODIPY 581/591 C11 dye (Invitrogen, D3861) (to detect lipid peroxidation) in medium for 30 min. Next, the cells were digested to obtain a cell suspension. Detection was performed by flow cytometer (BD FACS AriaIII, BD Biosciences).

### Measurement of GPX activity

Glutathione peroxidase (GPX) activity was measured using Beyotime’s Cellular Glutathione Peroxidase Assay Kit (S0056). The cell lysate was mixed with GPX detection working solution and reacted at 25 °C for 15 min. Reactions were initiated using a peroxide reagent (t-Bu-OOH) and absorbance was measured at 340 nm for 20 min (measured every minute). Calculate the activity value of glutathione peroxidase in the sample according to the OD value.

### MDA assay

Intracellular lipid peroxidation was detected using Beyotime’s MDA detection kit (S0131M). After the collected cells were lysed, the cell lysates were treated with MDA standard solution. Afterward, mix the above liquid with the thiobarbituric acid (TBA) working solution. The absorbance of the MDA-TBA complex was measured at 532 nm, and the content of cellular MDA was calculated.

### Analysis of GSH and GSSG

Beyotime’s GSH and GSSG kit (S0053) was used to measure intracellular glutathione (GSH) and glutathione disulfide (GSSG) levels. After the cells were collected, the samples were deproteinized. Next, the samples were then subjected to two flash freeze-thaw cycles (liquid nitrogen and 37 °C water bath). After 5 min at 4 °C, centrifuge at 10,000 g for 10 minutes. Measure the concentrations of GSH and GSSG in the supernatant using a microplate reader at 412 nm.

### Antibodies

The experimental procedure was as described previously [36]. The antibodies used are as follows: RRM1 (600732-2-Ig, 1:1000, Proteintech, Rosemont, IL, USA), p53 (10442-1-AP, 1:1000, Proteintech, Rosemont, IL, USA), GPX4 (ab125066, 1:1000, Abcam, Cambridge, UK), MDM2 (#86934, 1:1000, Cell Signaling Technology, Danvers, MA, USA), USP11 (22340-1-AP, 1:1000, Proteintech, Rosemont, IL, USA), p53 (#2524, 1:1000, Cell Signaling Technology, Danvers, MA, USA), Ubiquitin (#3936, 1:1000, Cell Signaling Technology, Danvers, MA, USA), p21 (sc-6246, 1:5000, Santa Cruz, CA, USA), Puma (55120-1-Ap, 1:1000, Proteintech, Rosemont, IL, USA), cleaved caspase-9 (sc8355, 1:1000, Santa Cruz, CA, USA), caspase-7 (#9492 S, 1:1000, Cell Signaling Technology, Danvers, MA, USA), cleaved caspase-3 (MAB835, 1:1000, R&D Systems, Minneapolis, MN, USA), γH2AX (05-636, 1:1000, Sigma, Merck KGaA, Darmstadt, Germany), β-actin (T0022,1:3000, Affinity, San Francisco, CA, USA), and Histone H3 (AF0009, 1:1500, Beyotime, Shanghai, China).

### RNA sequencing

A total of 1 × 10^6^ cells were plated per well in a 10 cm dish. Both the control and RRM1 knockdown cell lines were treated with radiation (γ-ray, 5 Gy). After 24 hours, the total RNA extraction was used Trizol (Invitrogen, Shanghai, China). Samples were sent to Suzhou Jinweizhi Company for sequencing at Illumina Hiseq 4000 system. Pathways associated with radiation treatment were enriched using Gene Set Enrichment Analysis (GSEA) software.

### Immunofluorescence staining

Cells were fixed with 4% paraformaldehyde after a brief rinse with PBS. 0.2% Triton X-100 was used to permeabilize cells. Afterwards, cells were blocked for 2 hours. Next, cells were incubated overnight at 4 °C with primary antibodies diluted in blocking buffer. On the second day, after washing the cells with PBS, incubate the secondary antibody for 1 hour at room temperature in the dark. Nuclei were counterstained with DAPI.

### Statistical analysis

The experiments were performed three times independently. The mean plus or minus the standard error is a representation of statistical data. Two groups were compared by Student’s *t*-test, and multiple groups were compared by two-way ANOVA. *P* < 0.05 represents a significant difference. *P < 0.05, **P < 0.01, ***P < 0.001, ****P < 0.0001, ns = not significant.

## Supplementary information

Supplementary Material

Original images for western blots

## Data Availability

All data are available in the main text or the supplementary materials.
