# Supplementary material for: Knockdown of RRM1 in tumor cells promotes radio-/chemotherapy induced ferroptosis by regulating p53 ubiquitination and p21-GPX4 signaling axis
Source: Cell Death Discov. 2022 Aug 1;8:343. doi: 10.1038/s41420-022-01140-z (PMC9343379; doi:10.1038/s41420-022-01140-z)
Supplement: Supplementary file 1 — Supplementary Material [file 41420_2022_1140_MOESM1_ESM.doc]

**
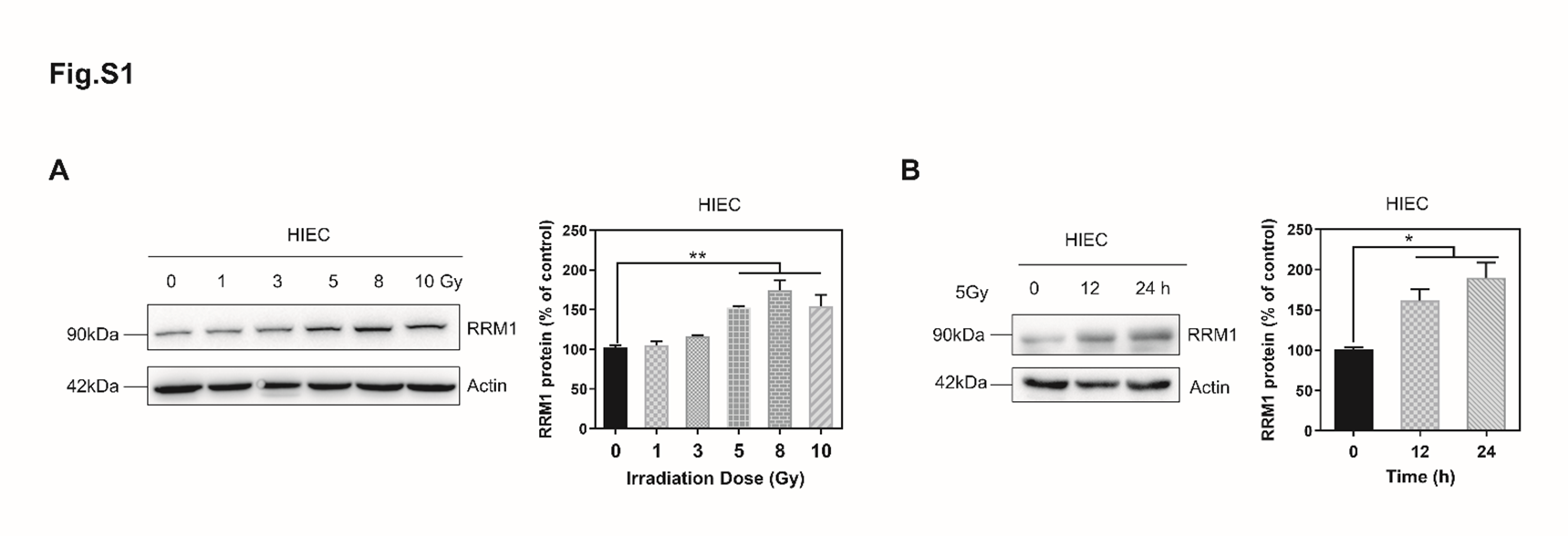
**

**Figure S1.** **(A)** HIEC cells were irradiated with different doses of γ-radiation, and the protein levels of RRM1 were analyzed by western blotting 24 h later. **(B)** The expression of RRM1 in HIEC cells at different time points after 5 Gy γ-radiation. * *P* < 0.05, ** *P* < 0.01.

**
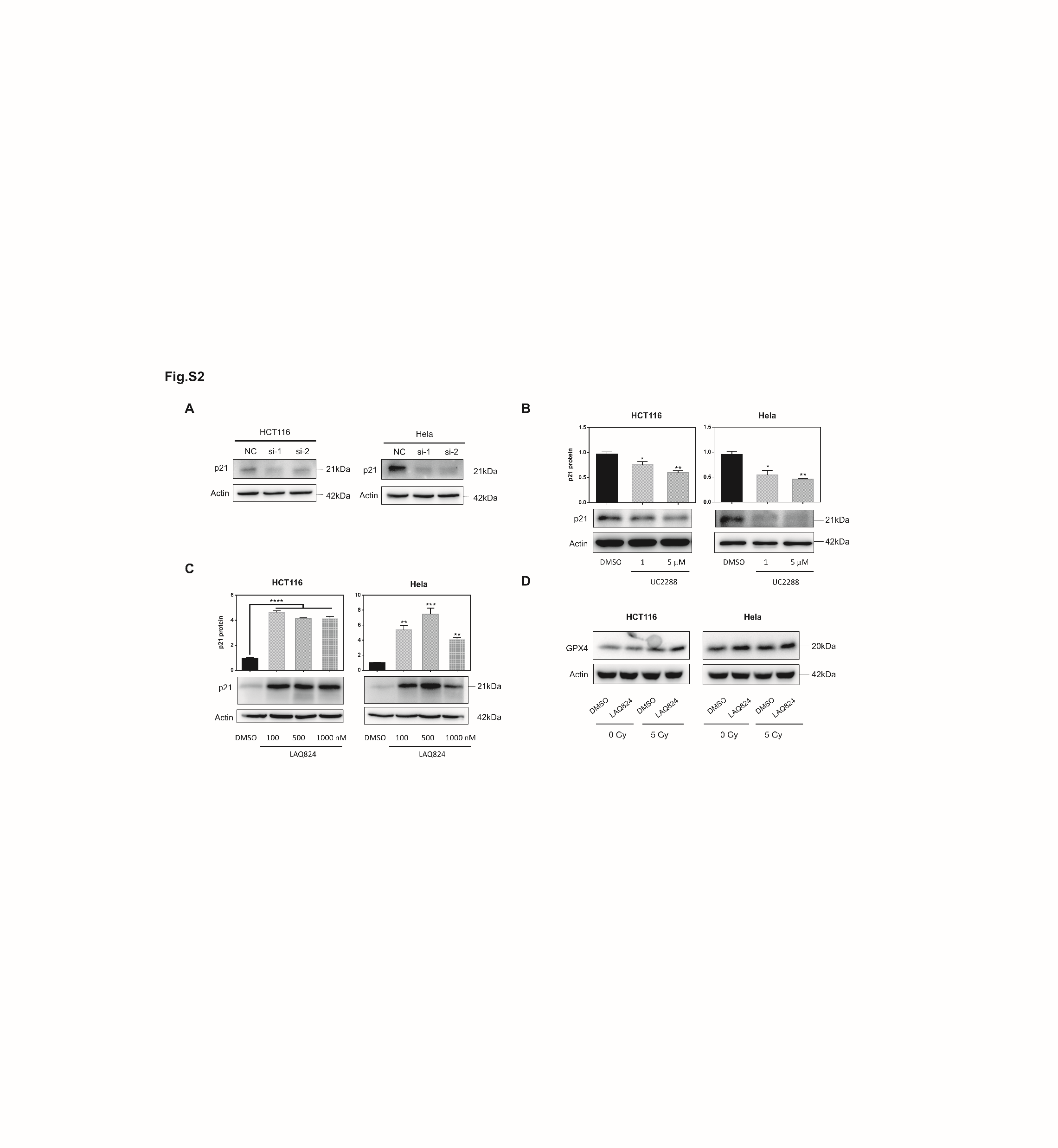
**

**Figure S2.** Effects of using UC2288 and LAQ824 on the expression level of p21 and GPX4. **(A-B)** Immunoblotting analysis to detect the inhibition efficiency of p21. (si-1/2 stands for two interference segments). **(C)** The activation of p21 in different concentrations of LAQ824 was detected by western blot analysis. **(D)** The expression of GPX4 was detected in control and 500 nM LAQ824 treated cells at 24 h after 5 Gy Gamma-ray irradiations. Actin was used as internal control. Data were pooled from three independent experiments and the results were represented as mean ± SD. * *P* < 0.05, ** *P* < 0.01, *** *P* < 0.001, **** *P* < 0.0001.

**
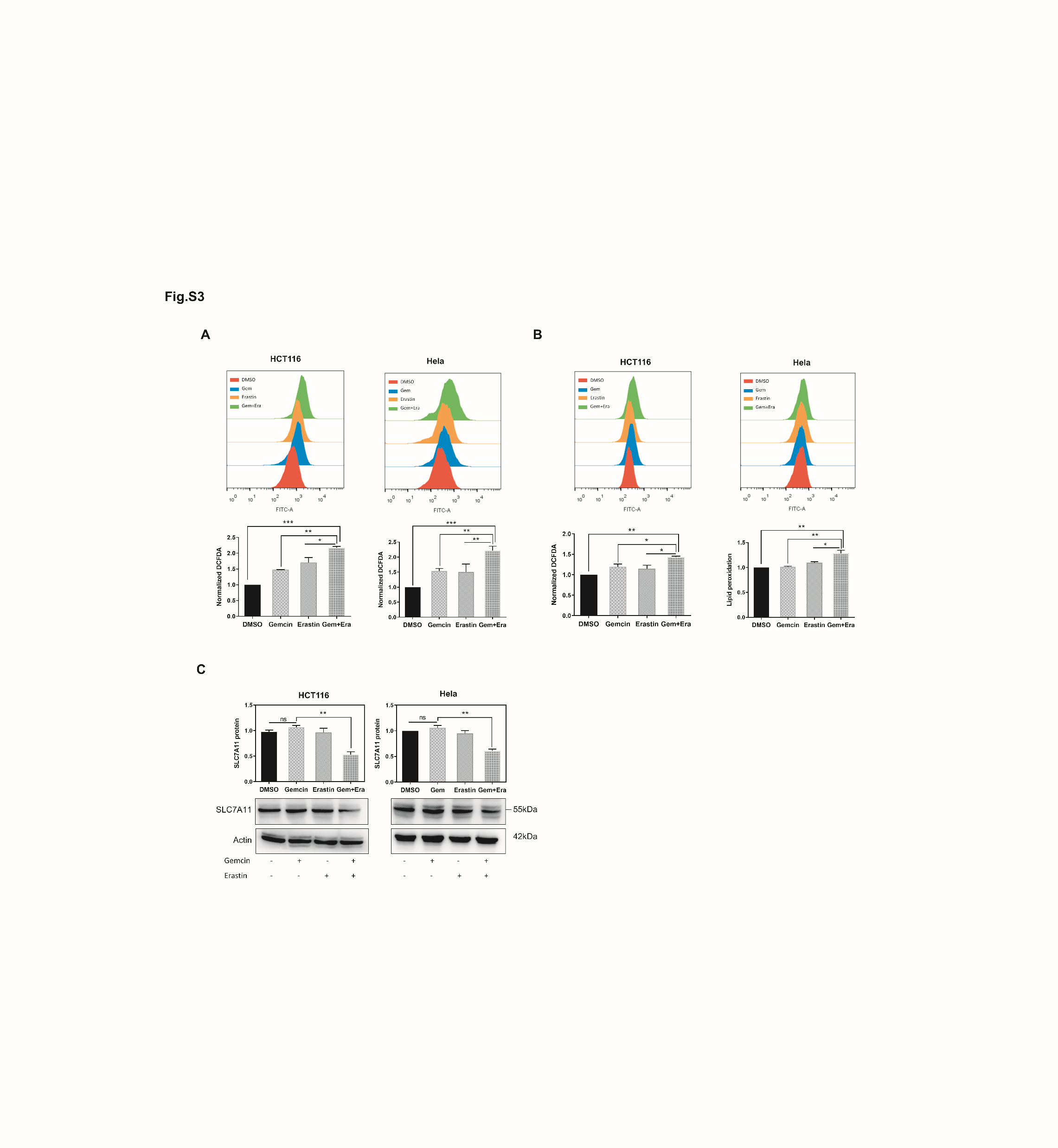
**

**Figure S3.** Effect of combination of gemcitabine and erastin on cells. **(A-C)** Total ROS levels (A), lipid peroxidation (B) and SLC7A11 expression (C) were detected in HCT116 and Hela cells at 24 h after exposure to 10 nM gemcitabine (Gem) and 50 µm erastin (Era). Actin was used as internal control. Data were pooled from three independent experiments and the results were represented as mean ± SD. * *P* < 0.05, ** *P* < 0.01, ns = not significant.

**Table S1.** **siRNA sequences of siRNA-NC and siRNA-p21**

| **Gene** | **Forward primer** | **Reverse primer** |
| --- | --- | --- |
| NC | 5'-UUCUCCGAACGUGUCACGUTT-3' | 5'-ACGUGACACGUUCGGAGAATT-3' |
| si-p21-1 | 5'-CUUCGACUUUGUCACCGAGTT-3' | 5'-CUCGGUGACAAAGUCGAAGUU-3' |
| si-p21-2 | 5'-GAUGGAACUUCGACUUUGUTT-3' | 5'-ACAAAGUCGAAGUUCCAUCTT-3' |

**Table S2.** **shRNA sequence of RRM1**

| **Gene** | **Sequence** |
| --- | --- |
| shRNA-1 | F: 5'-CCGGCCAATCCAGTTCACTCTAAATCTCGAGATTTAGAGTGAACTGGATTGGTTTTTG-3'  R: 5'-AATTCAAAAACCAATCCAGTTCACTCTAAATCTCGAGATTTAGAGTGAACTGGATTGG-3' |
| shRNA-2 | F: 5'-CCGGCCTGCTCAGATCACCATGAAACTCGAGTTTCATGGTGATCTGAGCAGGTTTTTG-3'  R: 5'-AATTCAAAAACCTGCTCAGATCACCATGAAACTCGAGTTTCATGGTGATCTGAGCAGG-3' |
| shRNA-3 | F: 5'-CCGGCCCACAACTTTCTAGCTGTTTCTCGAGAAACAGCTAGAAAGTTGTGGGTTTTTG-3'  R: 5'-AATTCAAAAACCCACAACTTTCTAGCTGTTTCTCGAGAAACAGCTAGAAAGTTGTGGG-3' |

**Supplementary Materials and Methods**

**Transfection of Small Interfering RNA (siRNA) and shRNA Sequence**

The siRNA (si-RRM1, si-p21) and shRNA sequences were purchased from GenePharma (Shanghai, China) and Genewiz (Suzhou, China), and siRNA transfection was carried out using Lipofectamine 2000 (Thermo Fishier, Carlsbad, CA, USA) according to the manufacturer’s protocols. The siRNA sequences and shRNA sequences are listed in Tables S1 and S2 (Supplementary Materials).

**Lentivirus Production and Cell Culture**

To produce the lentivirus, HEK293T cells were transfected with the shRNA plasmids along with the helper plasmids psPAX2 and pMD2.G, with PEI (Thermo Fishier, Carlsbad, CA, USA) transfection reagent used as a lipid transport milieu. Lentivirus in the medium was obtained 48–72 h after transfection. We infected cells using respective lentiviral supernatants with 10 µg/mL polybrene (Sigma, Merck KGaA, Darmstadt, Germany) to generate HCT116 and Hela cells with reduced RRM1 expression or the vector control. Stable cell lines were obtained by culturing cells in the medium containing 1.5 mg/mL puromycin (Beyotime, Shanghai, China). Cells were all cultured in a 5% CO_2_-humidified incubator at 37℃.

**Immunoprecipitation assay**

Total cell lysates were prepared using RIPA Lysis Buffer (Beyotime, Shanghai, China) containing protease inhibitors. After centrifugation, the primary antibody was added to the supernatant and incubated at 4℃ for 12 h while gently stirring. Protein G Sepharose Bead Slurry (Beyotime, Shanghai, China) was then added to capture the protein complex. After incubation at 4℃ for 3 h with gentle agitation, the samples were centrifuged for 5 min at 4℃. The supernatant was discarded and the pellet was washed with RIPA Lysis Buffer. Finally, the immunoprecipitates were resuspended for Western blot analysis using an SDS-PAGE loading buffer.

**Reagents**

MTT (3-4,5-dimethyl-2-thiazolyl-2,5-diphenyl-2-H-tetrazolium bromide) was purchased from Sigma-Aldrich (Merck KGaA, Darmstadt, Germany). The ferroptosis inhibitor ferrostatin-1 was purchased from absin (Shanghai, China). The caspase inhibitor Z-VAD-FMK and proteasome inhibitor MG132 were purchased from TOPSCIENCE (Shanghai, China). Dimethyl sulfoxide (DMSO) and other chemicals were purchased from Sangon (Shanghai, China). Protein G Sepharose beads were purchased from Beyotime (Shanghai, China).

**Cell Viability Assay**

Cell viability was measured using the MTT assay. Briefly, the treated cells were cultured in a 96-well plate; then, after incubation for 24 h at 37 ℃, 5 µL of MTT reagent (5 mg/mL in PBS) was added to each well, followed by incubation for 4 h. After that, the cells were incubated with 150 µL of DMSO for 30 min to dissolve the crystals. The absorbance of each well at 490 nm was determined using a Multimode Reader of SpectraMax M2 (Molecular Devices, Sunnyvale, CA, USA).

**Colony Formation Assay**

A total of 800 cells were seeded in a 60 mm dish. After irradiation, the dishes were incubated for two weeks at 37℃ in a 5% CO_2_ incubator for 20 days. Then, the dishes were washed with PBS, fixed with a solution containing methanol/acetic acid (v/v = 9:1) for 30 min, and subsequently stained with crystal violet for 30 min. The colonies containing more than 50 cells per colony were scored and plotted.
