# Supplementary material for: Knockdown of RRM1 in tumor cells promotes radio-/chemotherapy induced ferroptosis by regulating p53 ubiquitination and p21-GPX4 signaling axis
Source: Cell Death Discov. 2022 Aug 1;8:343. doi: 10.1038/s41420-022-01140-z (PMC9343379; doi:10.1038/s41420-022-01140-z)
Supplement: Supplementary file 2 — Original images for western blots [file 41420_2022_1140_MOESM2_ESM.pdf]

Figure 1B

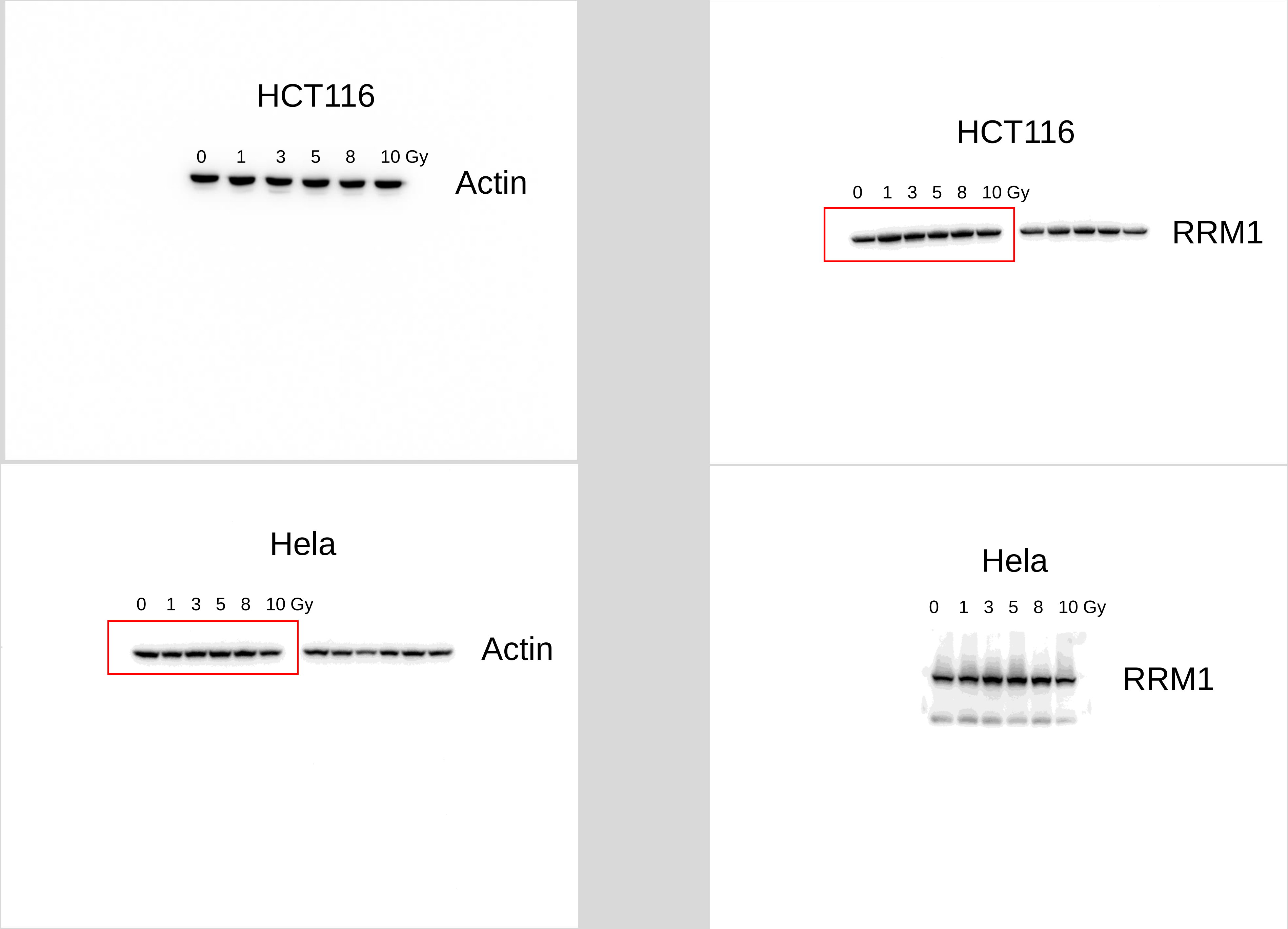
HCT116
0 1 3 5 8 10 Gy
Actin
HCT116
0 1 3 5 8 10 Gy
RRM1
Hela
0 1 3 5 8 10 Gy
Actin
Hela
0 1 3 5 8 10 Gy
RRM1

Figure 1C

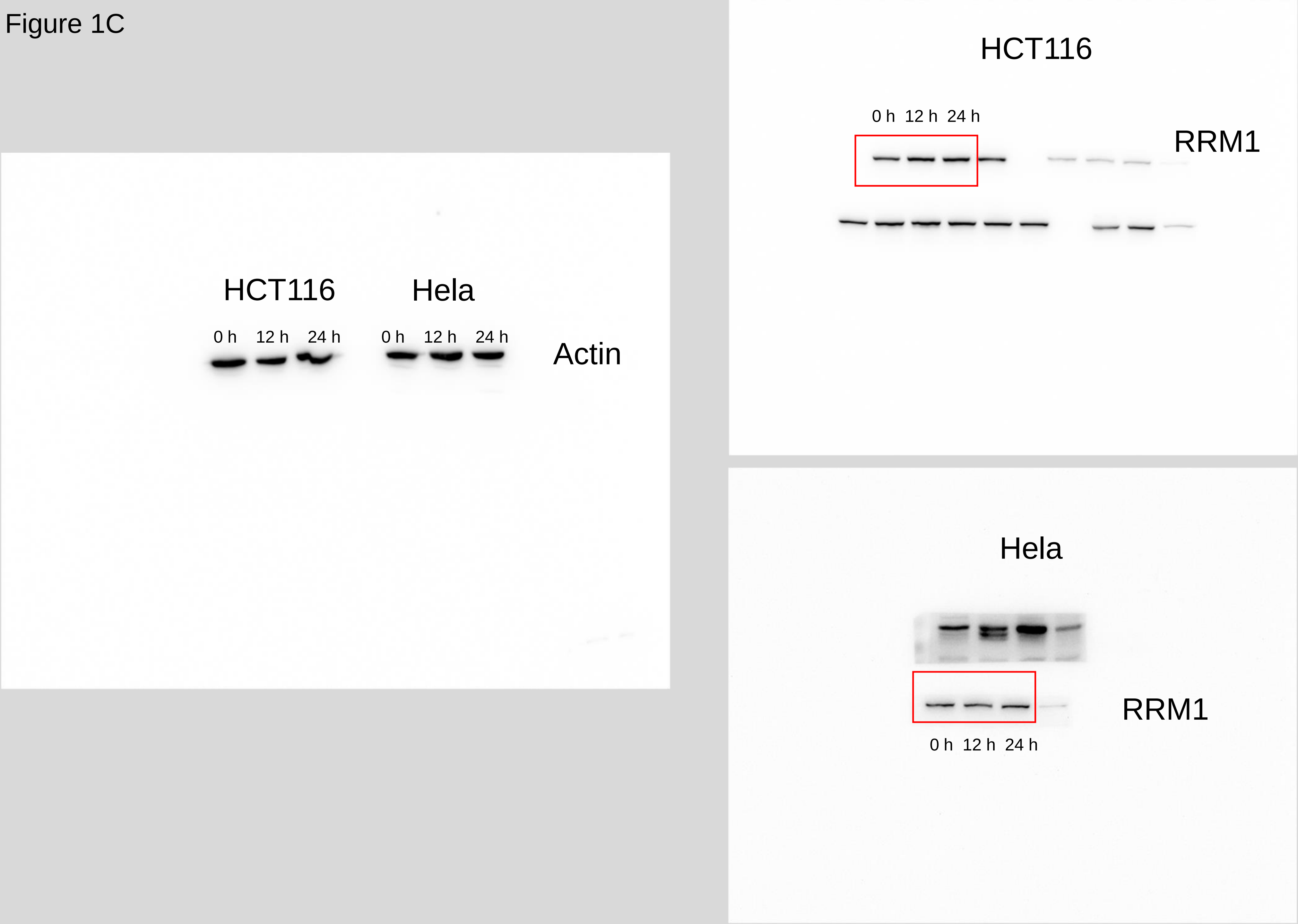
HCT116
Hela
0 h
12 h
24 h
0 h
12 h
24 h
Actin
HCT116
0 h
12 h
24 h
RRM1
Hela
RRM1
0 h
12 h
24 h

Figure 1D

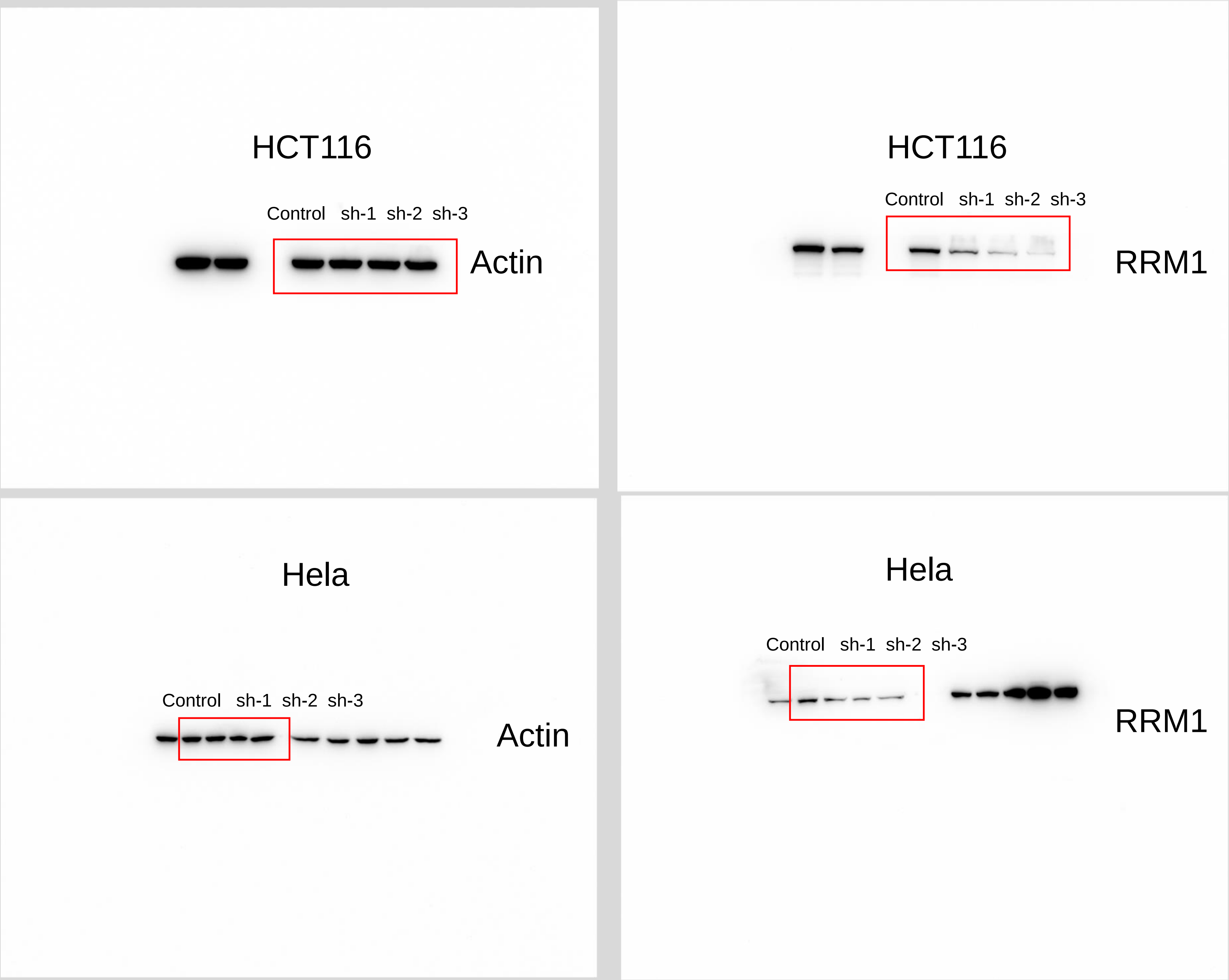
HCT116
Control sh-1 sh-2 sh-3
Actin
HCT116
Control sh-1 sh-2 sh-3
RRM1
Hela
Control sh-1 sh-2 sh-3
Actin
Hela
Control sh-1 sh-2 sh-3
RRM1

Figure 2E

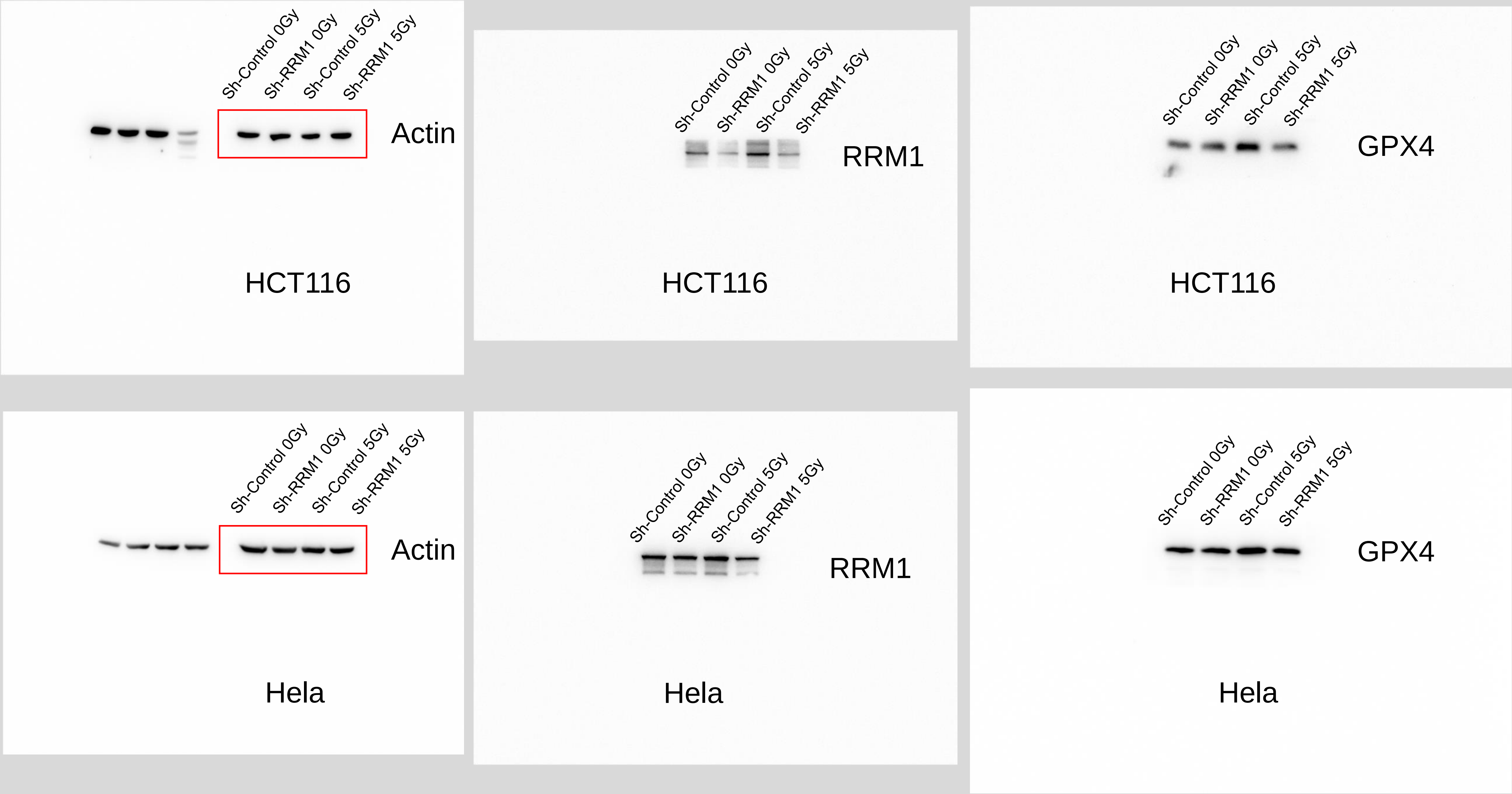
Sh-Control 0Gy
Sh-RRM1 0Gy
Sh-Control 5Gy
Sh-RRM1 5Gy
Actin
HCT116
RRM1
HCT116
GPX4
HCT116
Actin
Hela
RRM1
Hela
GPX4
Hela

Figure 3A

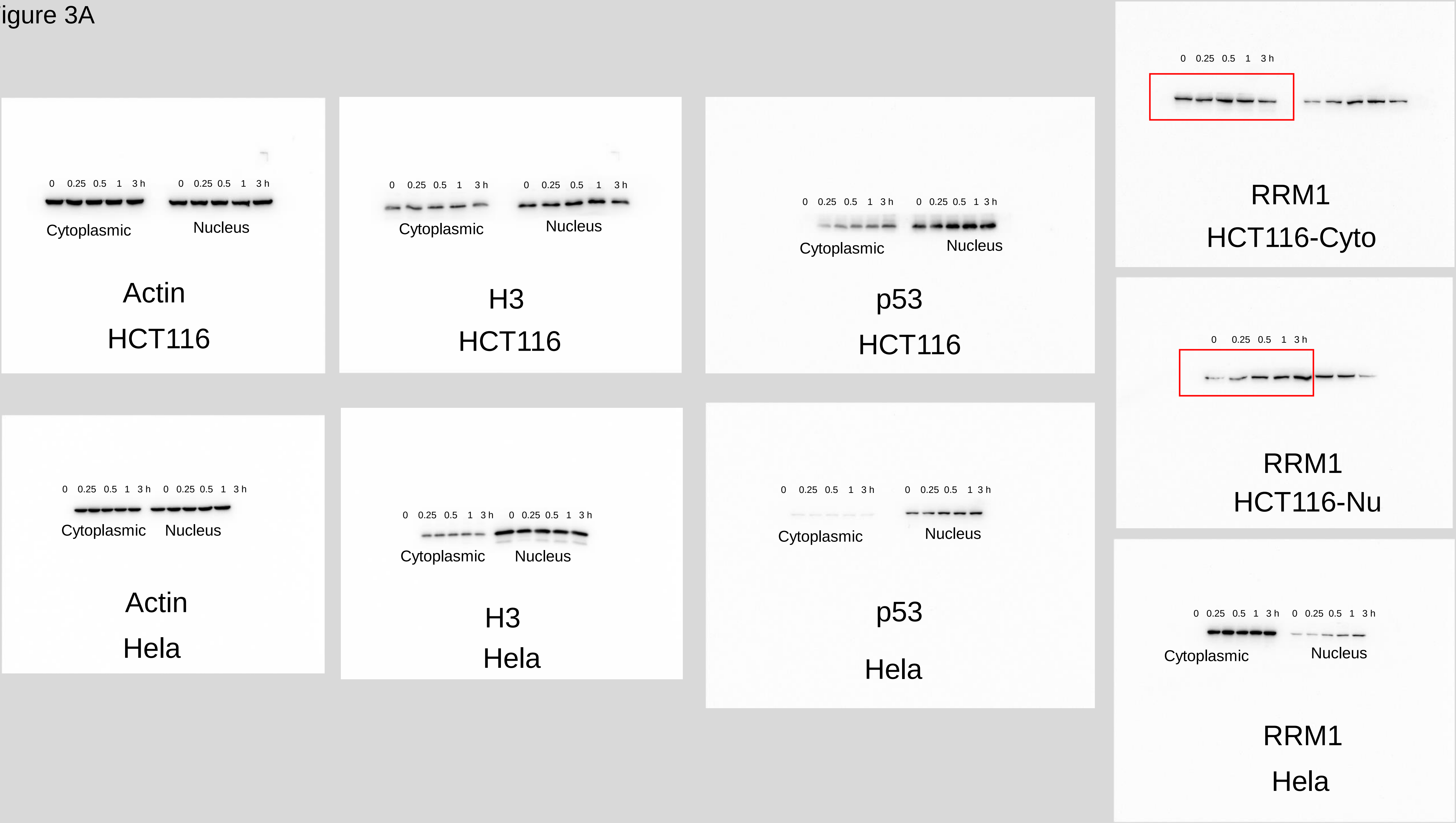
0 0.25 0.5 1 3 h 0 0.25 0.5 1 3 h
Cytoplasmic
Nucleus
Actin
HCT116
0 0.25 0.5 1 3 h 0 0.25 0.5 1 3 h
Cytoplasmic
Nucleus
H3
HCT116
0 0.25 0.5 1 3 h 0 0.25 0.5 1 3 h
Cytoplasmic
Nucleus
p53
HCT116
0 0.25 0.5 1 3 h
RRM1
HCT116-Cyto
0 0.25 0.5 1 3 h 0 0.25 0.5 1 3 h
Cytoplasmic
Nucleus
Actin
Hela
0 0.25 0.5 1 3 h 0 0.25 0.5 1 3 h
Cytoplasmic
Nucleus
H3
Hela
0 0.25 0.5 1 3 h 0 0.25 0.5 1 3 h
Cytoplasmic
Nucleus
p53
Hela
0 0.25 0.5 1 3 h
RRM1
HCT116-Nu
0 0.25 0.5 1 3 h 0 0.25 0.5 1 3 h
Cytoplasmic
Nucleus
RRM1
Hela

Figure 3B

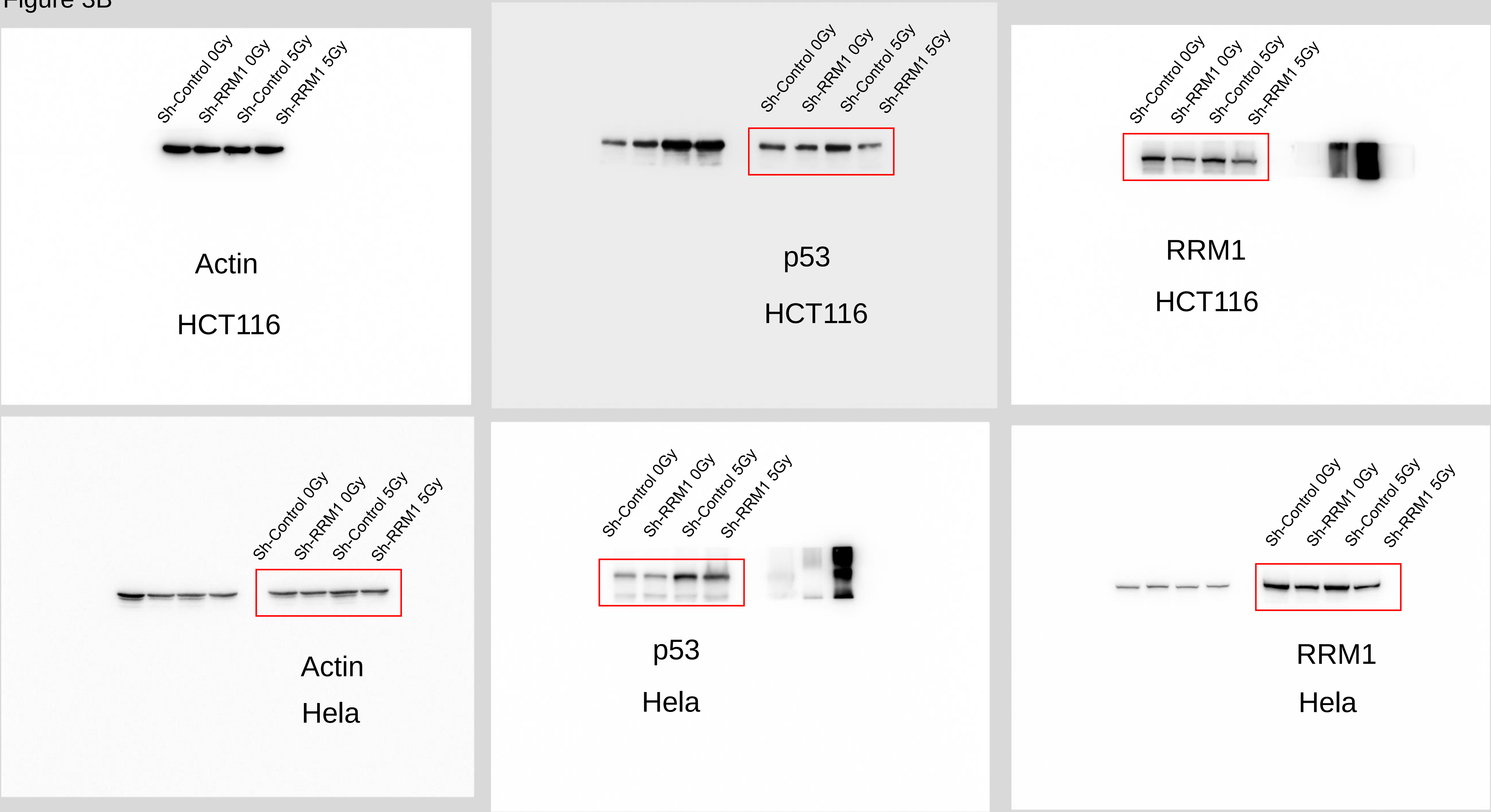

Sh-Control 0Gy
Sh-RRM1 0Gy
Sh-Control 5Gy
Sh-RRM1 5Gy
Actin
HCT116
Sh-Control 0Gy
Sh-RRM1 0Gy
Sh-Control 5Gy
Sh-RRM1 5Gy
p53
HCT116
Sh-Control 0Gy
Sh-RRM1 0Gy
Sh-Control 5Gy
Sh-RRM1 5Gy
RRM1
HCT116
Sh-Control 0Gy
Sh-RRM1 0Gy
Sh-Control 5Gy
Sh-RRM1 5Gy
Actin
Hela
Sh-Control 0Gy
Sh-RRM1 0Gy
Sh-Control 5Gy
Sh-RRM1 5Gy
p53
Hela
Sh-Control 0Gy
Sh-RRM1 0Gy
Sh-Control 5Gy
Sh-RRM1 5Gy
RRM1
Hela

Figure 3C

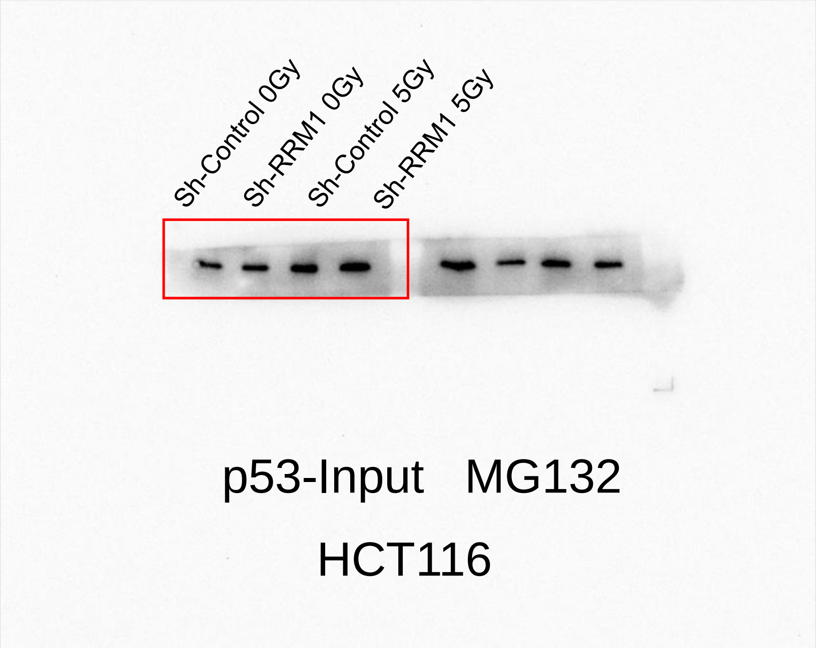
Sh-Control 0Gy
Sh-RRM1 0Gy
Sh-Control 5Gy
Sh-RRM1 5Gy
p53-Input
MG132
HCT116

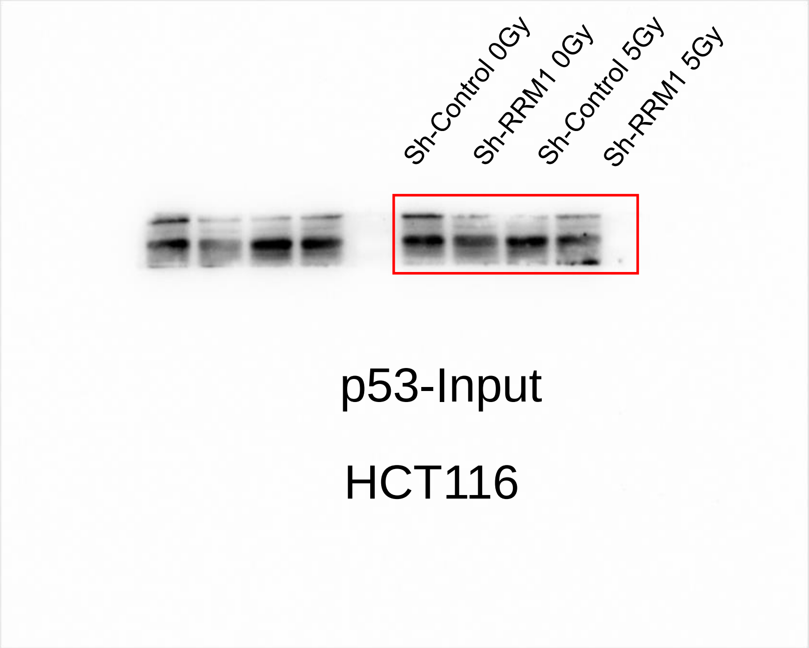
Sh-Control 0Gy
Sh-RRM1 0Gy
Sh-Control 5Gy
Sh-RRM1 5Gy
p53-Input
HCT116

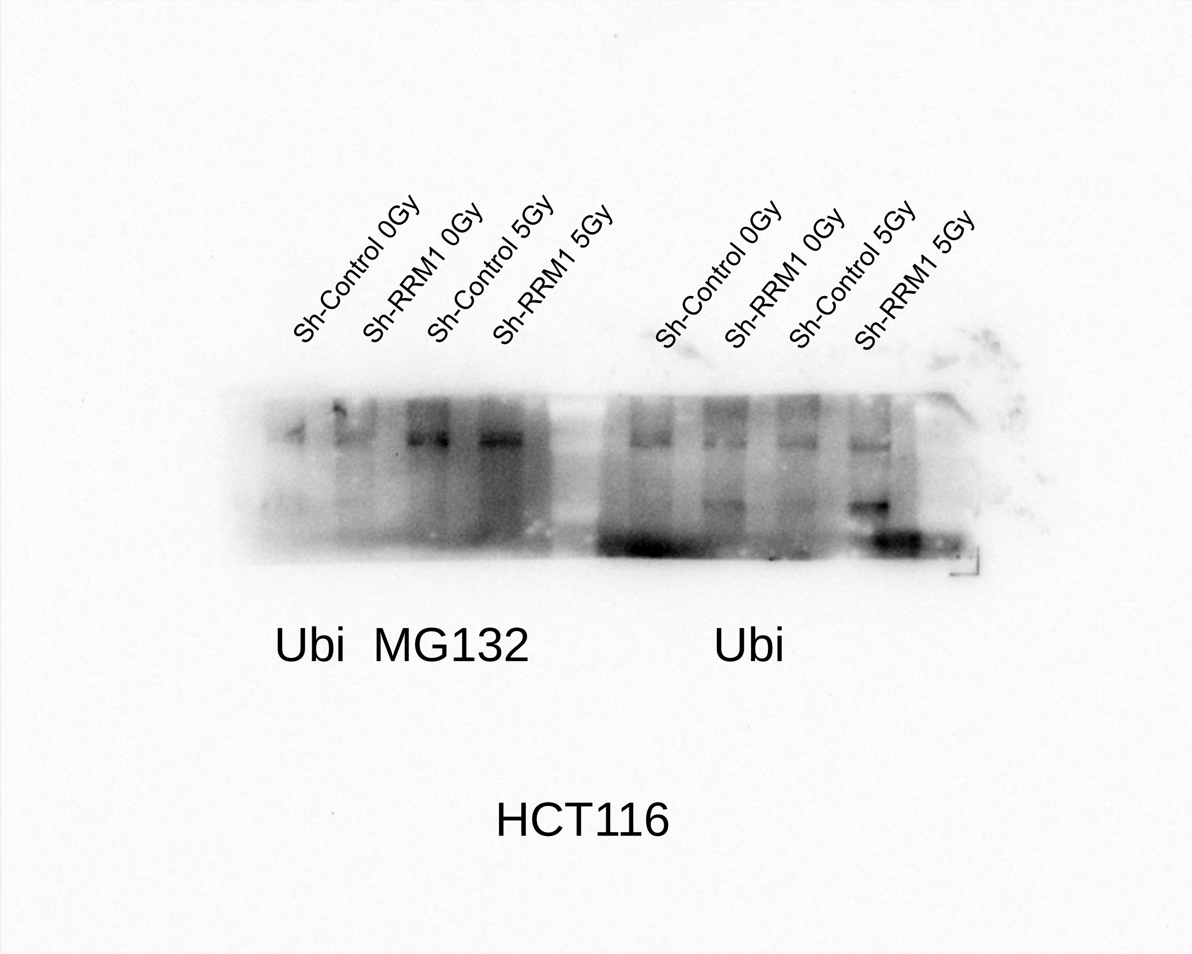
Sh-Control 0Gy
Sh-RRM1 0Gy
Sh-Control 5Gy
Sh-RRM1 5Gy
Sh-Control 0Gy
Sh-RRM1 0Gy
Sh-Control 5Gy
Sh-RRM1 5Gy
Ubi
MG132
Ubi
HCT116

Figure 3D

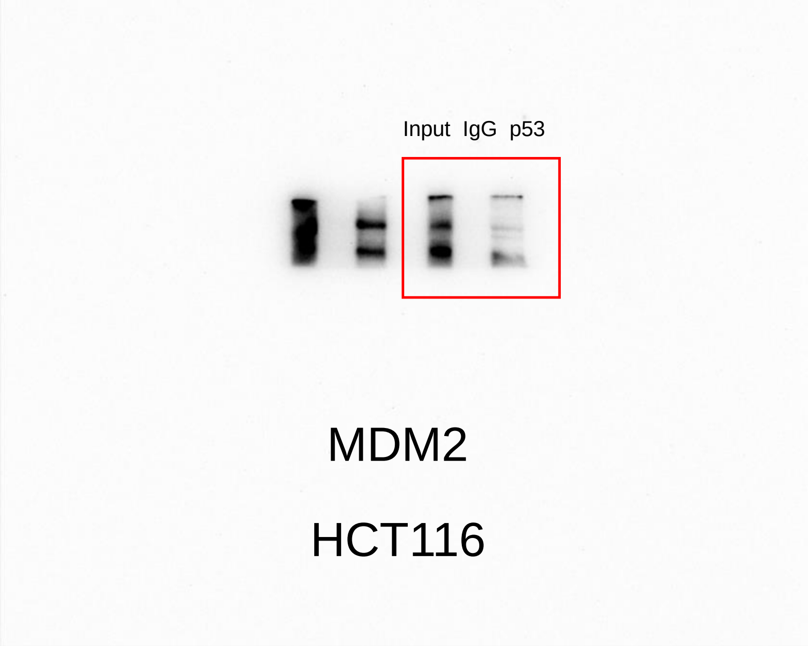
Input IgG p53
MDM2
HCT116

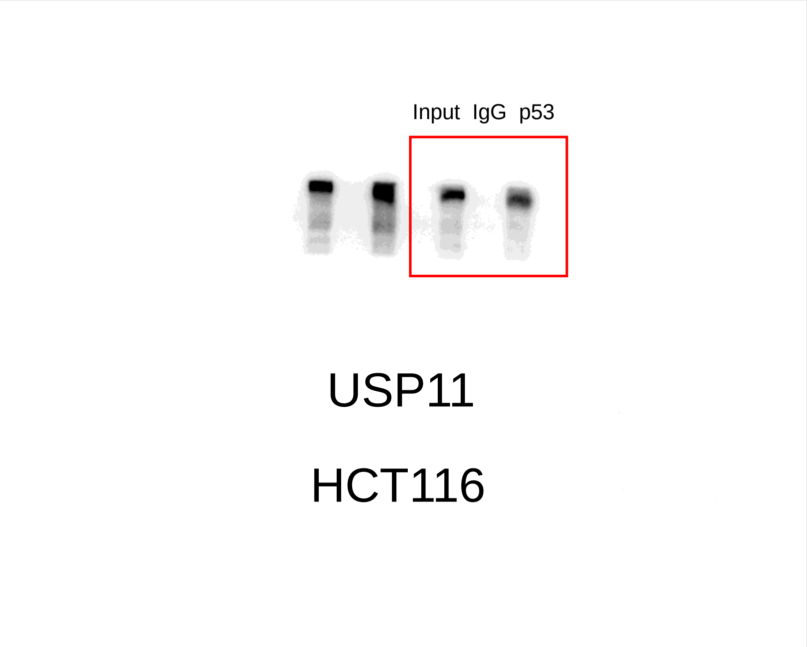
Input IgG p53
USP11
HCT116

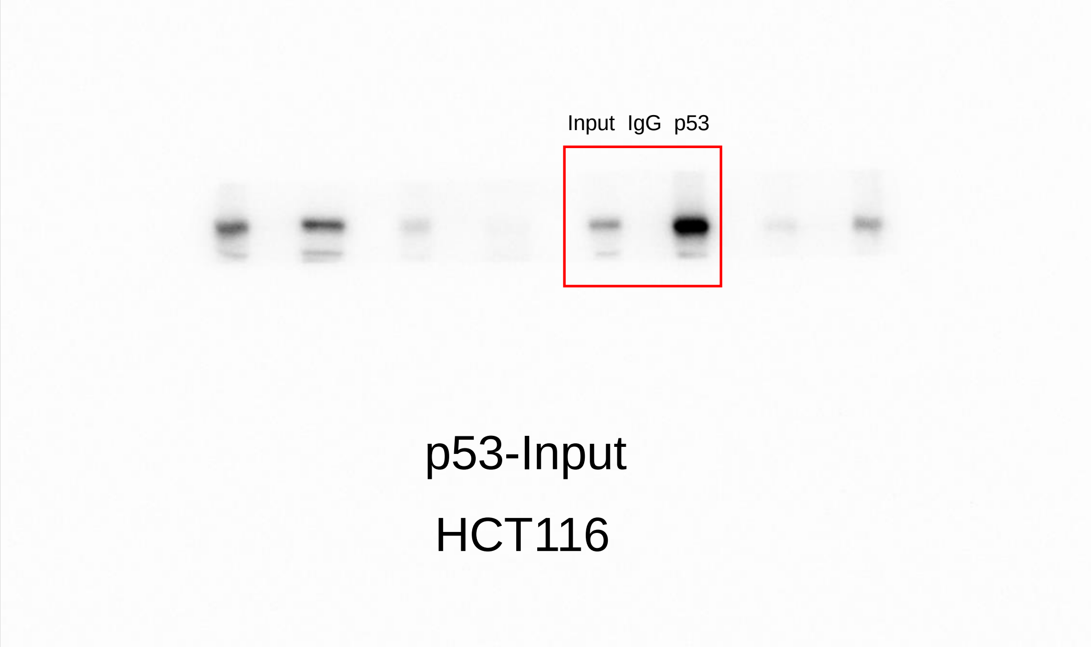
Input IgG p53
p53-Input
HCT116

Figure 3F

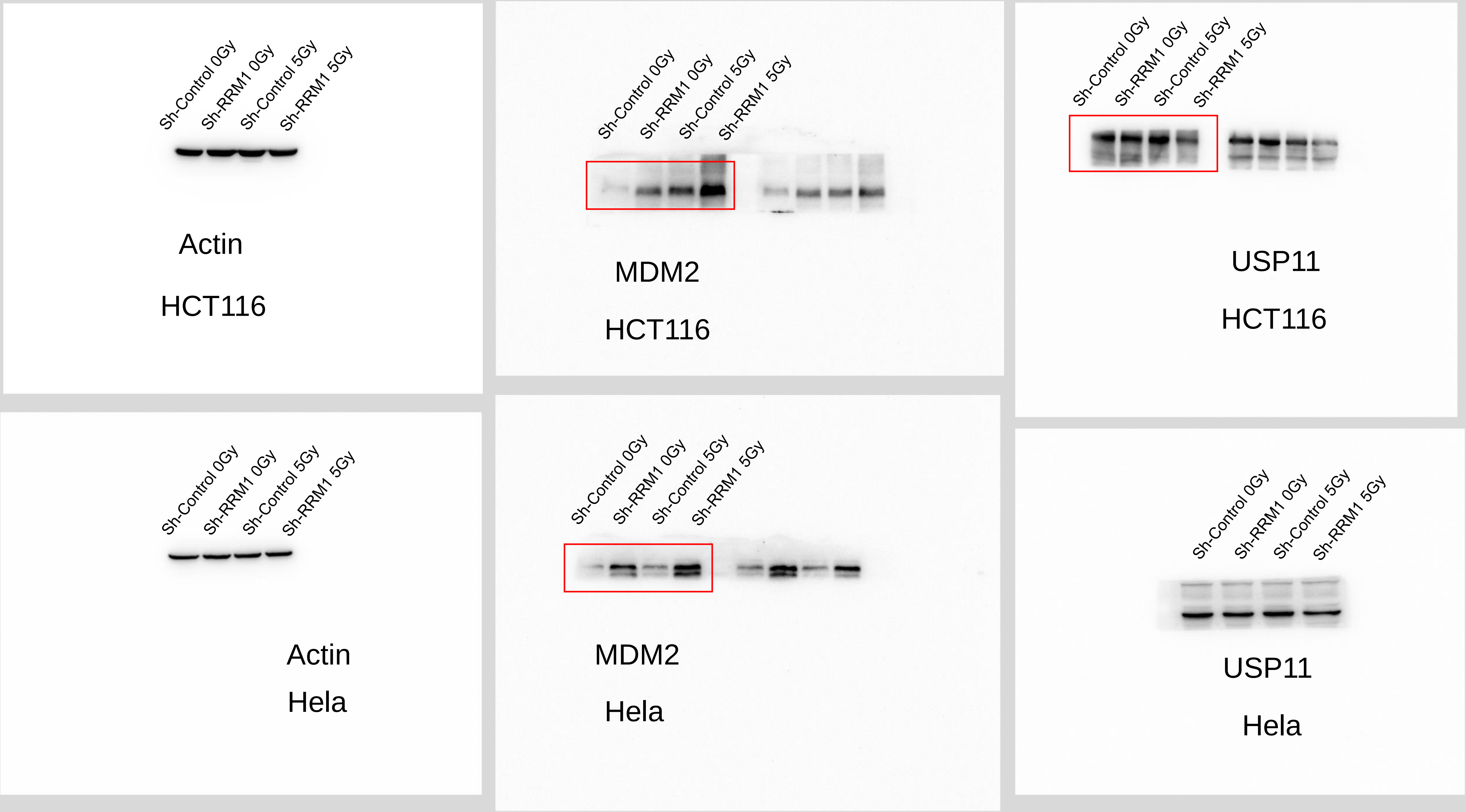
Sh-Control 0Gy
Sh-RRM1 0Gy
Sh-Control 5Gy
Sh-RRM1 5Gy
Actin
HCT116
Sh-Control 0Gy
Sh-RRM1 0Gy
Sh-Control 5Gy
Sh-RRM1 5Gy
MDM2
HCT116
Sh-Control 0Gy
Sh-RRM1 0Gy
Sh-Control 5Gy
Sh-RRM1 5Gy
USP11
HCT116
Sh-Control 0Gy
Sh-RRM1 0Gy
Sh-Control 5Gy
Sh-RRM1 5Gy
Actin
Hela
Sh-Control 0Gy
Sh-RRM1 0Gy
Sh-Control 5Gy
Sh-RRM1 5Gy
MDM2
Hela
Sh-Control 0Gy
Sh-RRM1 0Gy
Sh-Control 5Gy
Sh-RRM1 5Gy
USP11
Hela

Figure 3G

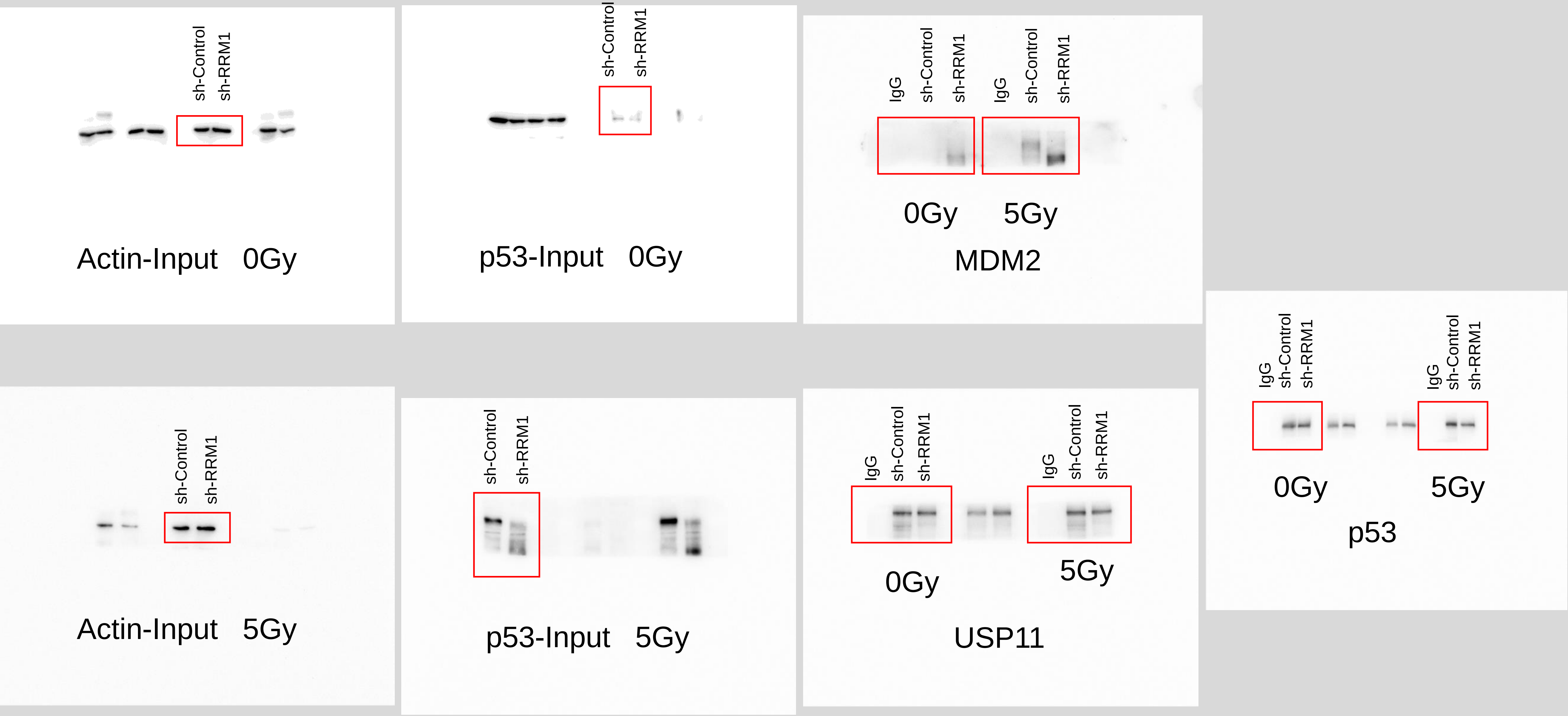
sh-Control
sh-RRM1
Actin-Input 0Gy
sh-Control
sh-RRM1
p53-Input 0Gy
IgG
sh-Control
sh-RRM1
IgG
sh-Control
sh-RRM1
0Gy
5Gy
MDM2
IgG
sh-Control
sh-RRM1
IgG
sh-Control
sh-RRM1
0Gy
5Gy
p53
sh-Control
sh-RRM1
Actin-Input 5Gy
sh-Control
sh-RRM1
p53-Input 5Gy
IgG
sh-Control
sh-RRM1
IgG
sh-Control
sh-RRM1
0Gy
5Gy
USP11

Figure 3J

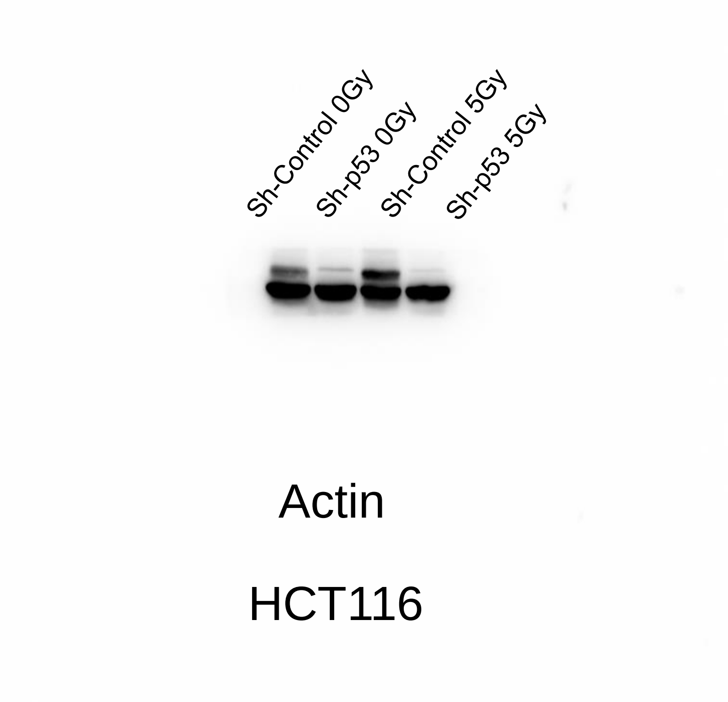
Sh-Control 0Gy
Sh-p53 0Gy
Sh-Control 5Gy
Sh-p53 5Gy
Actin
HCT116

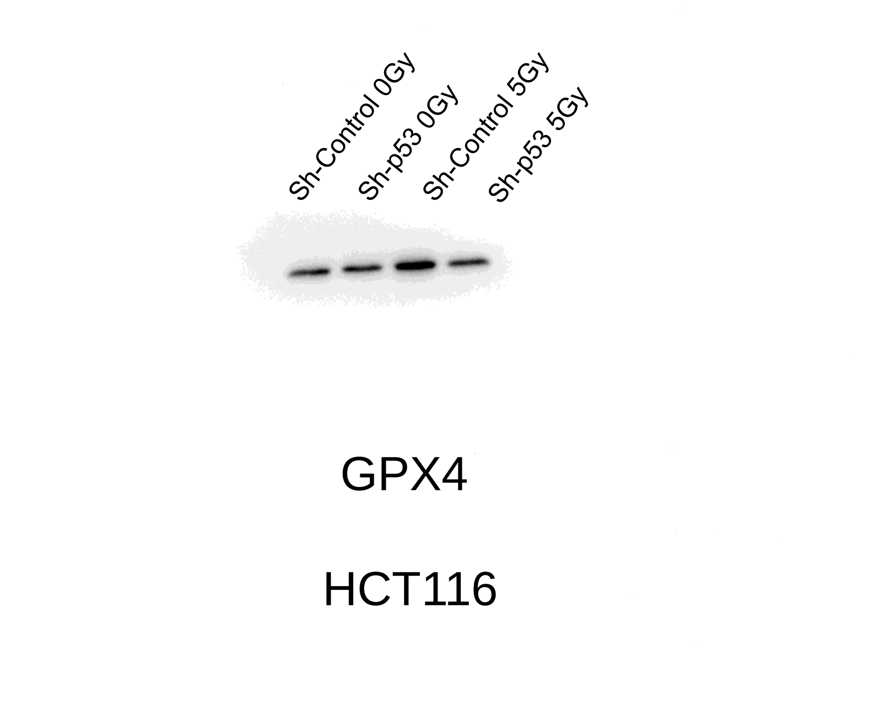
Sh-Control 0Gy
Sh-p53 0Gy
Sh-Control 5Gy
Sh-p53 5Gy
GPX4
HCT116

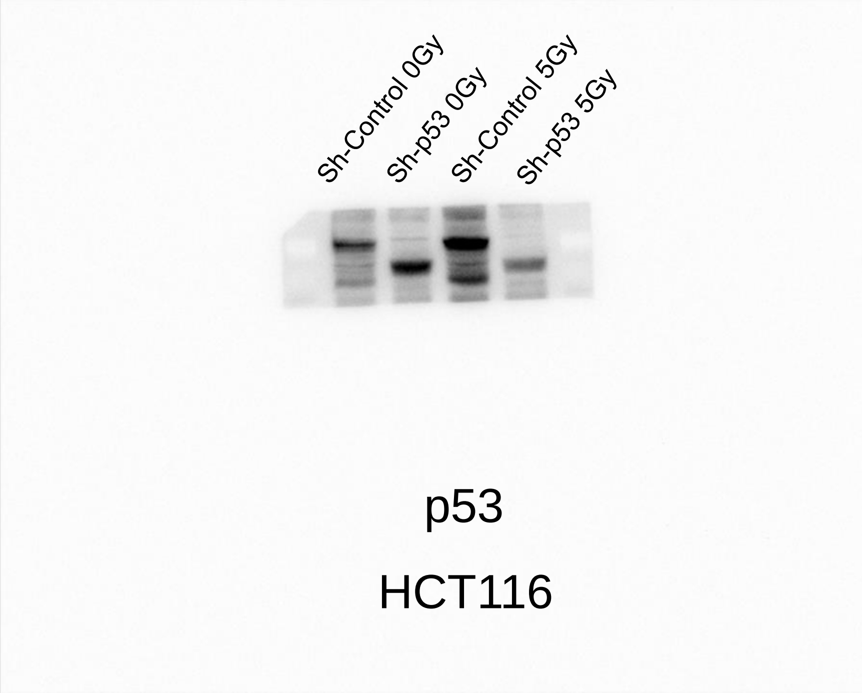
Sh-Control 0Gy
Sh-p53 0Gy
Sh-Control 5Gy
Sh-p53 5Gy
p53
HCT116

Figure 4A

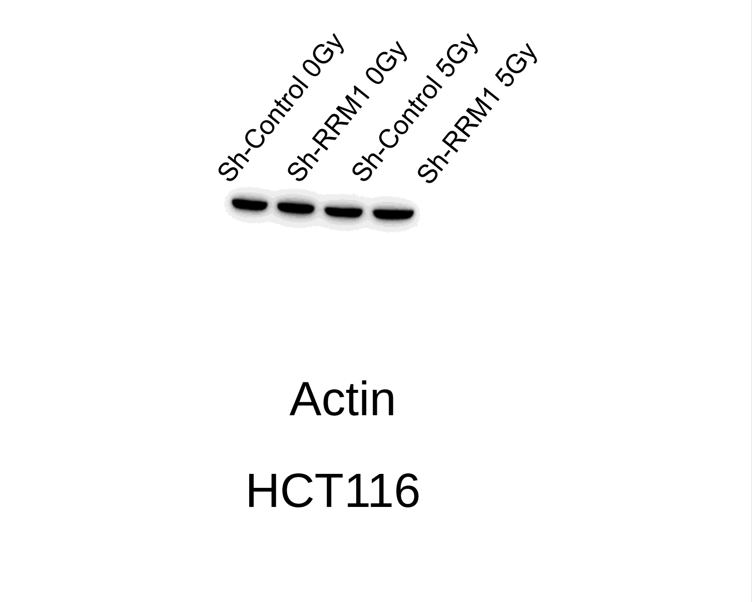

Sh-Control 0Gy
Sh-RRM1 0Gy
Sh-Control 5Gy
Sh-RRM1 5Gy
Actin
HCT116

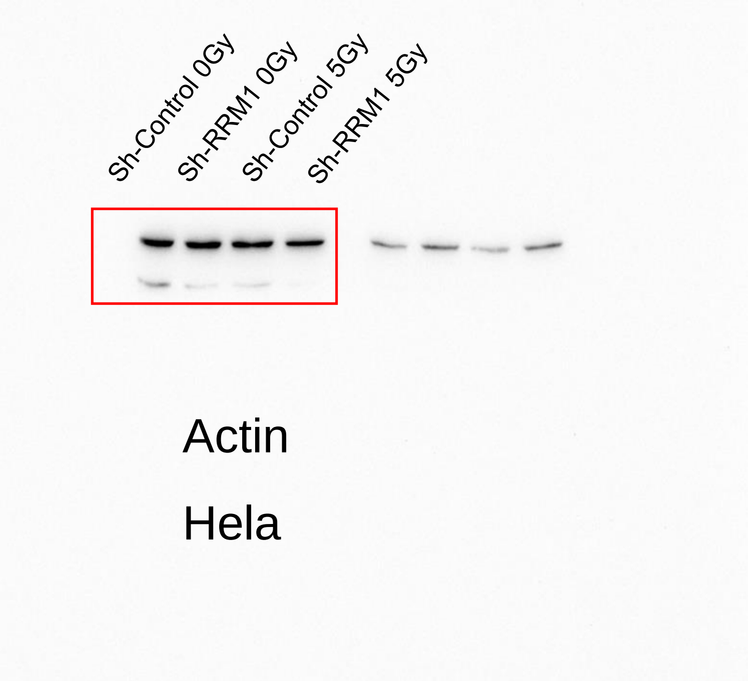

Sh-Control 0Gy
Sh-RRM1 0Gy
Sh-Control 5Gy
Sh-RRM1 5Gy
Actin
Hela

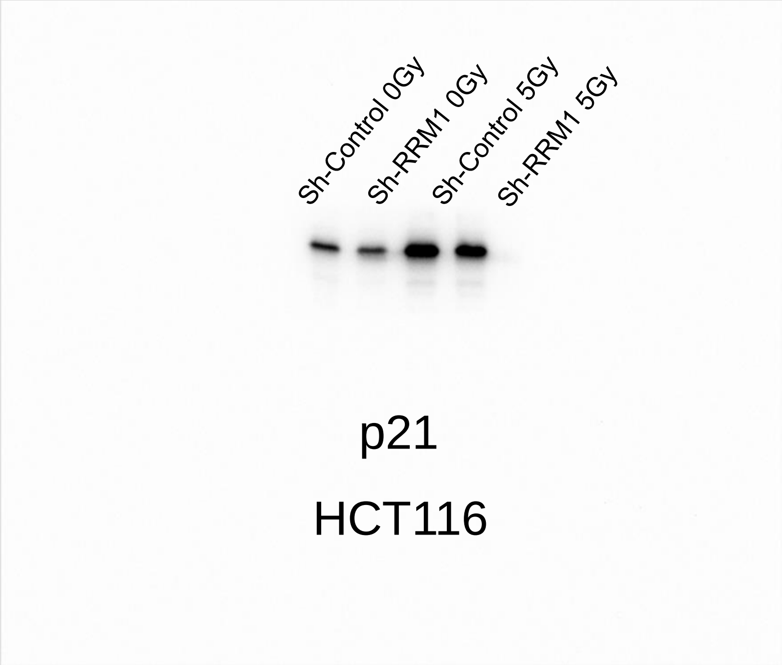

Sh-Control 0Gy
Sh-RRM1 0Gy
Sh-Control 5Gy
Sh-RRM1 5Gy
p21
HCT116

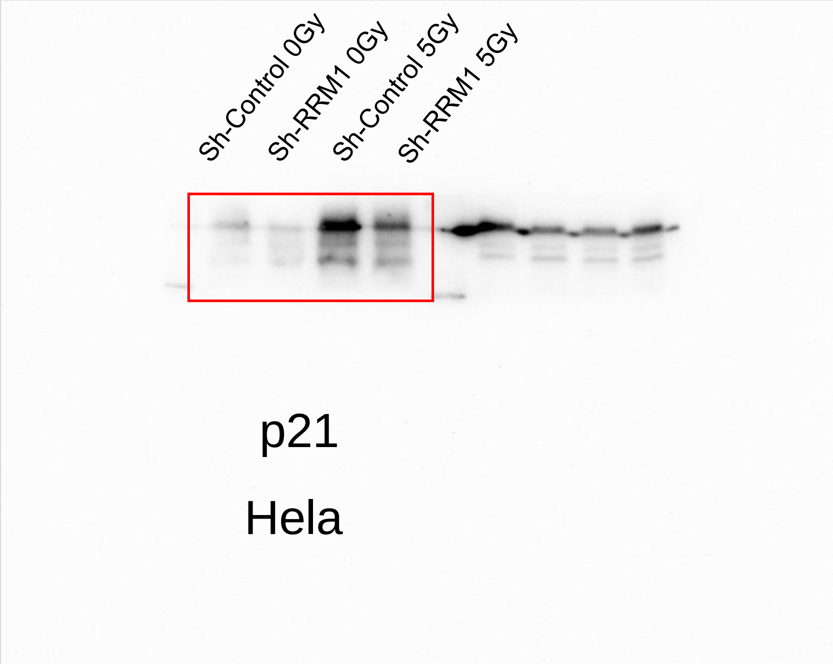

Sh-Control 0Gy
Sh-RRM1 0Gy
Sh-Control 5Gy
Sh-RRM1 5Gy
p21
Hela

Figure 4B

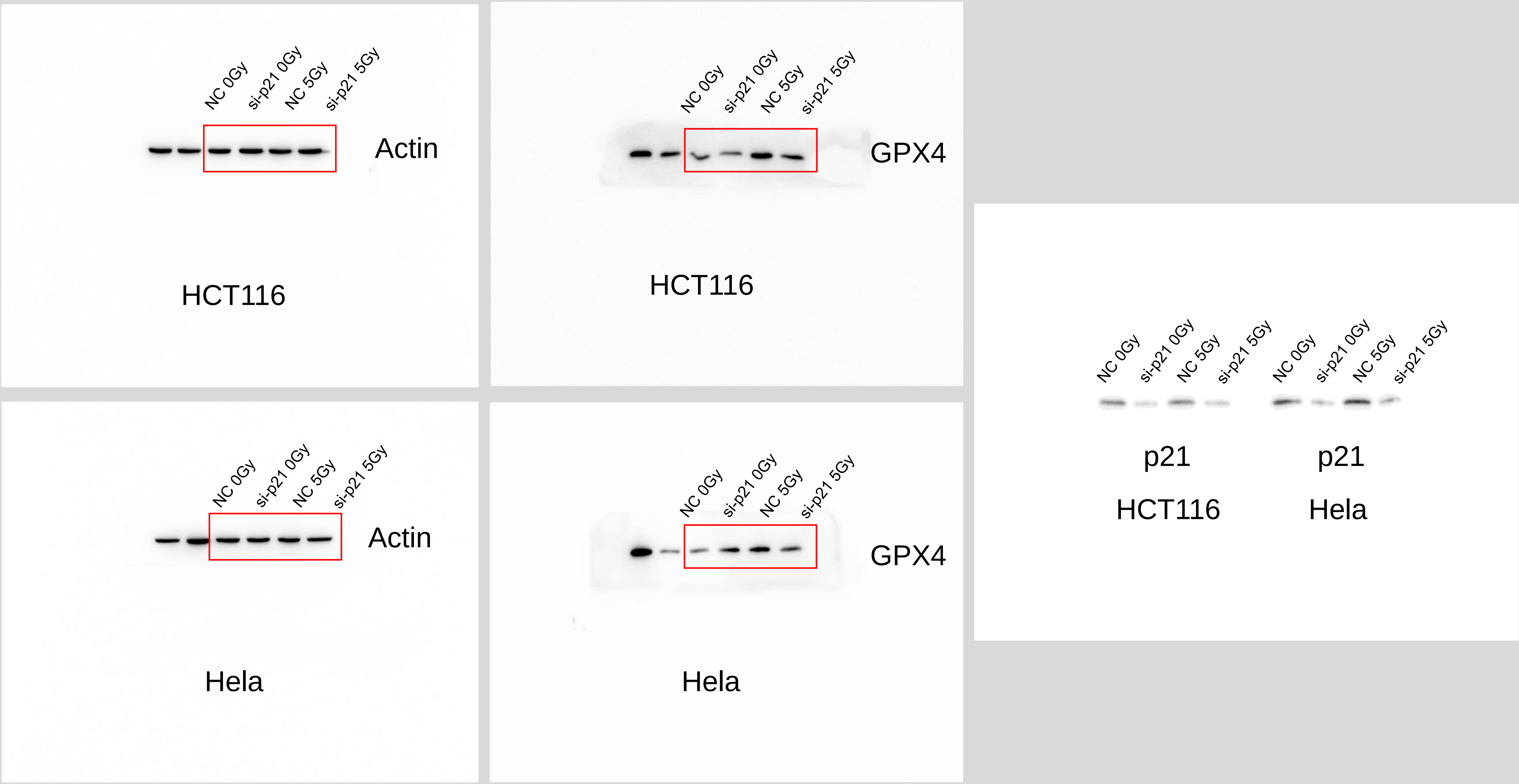
NC 0Gy
si-p21 0Gy
NC 5Gy
si-p21 5Gy
Actin
HCT116
NC 0Gy
si-p21 0Gy
NC 5Gy
si-p21 5Gy
GPX4
HCT116
NC 0Gy
si-p21 0Gy
NC 5Gy
si-p21 5Gy
Actin
Hela
NC 0Gy
si-p21 0Gy
NC 5Gy
si-p21 5Gy
GPX4
Hela
NC 0Gy
si-p21 0Gy
NC 5Gy
si-p21 5Gy
NC 0Gy
si-p21 0Gy
NC 5Gy
si-p21 5Gy
p21
p21
HCT116
Hela

Figure 4G

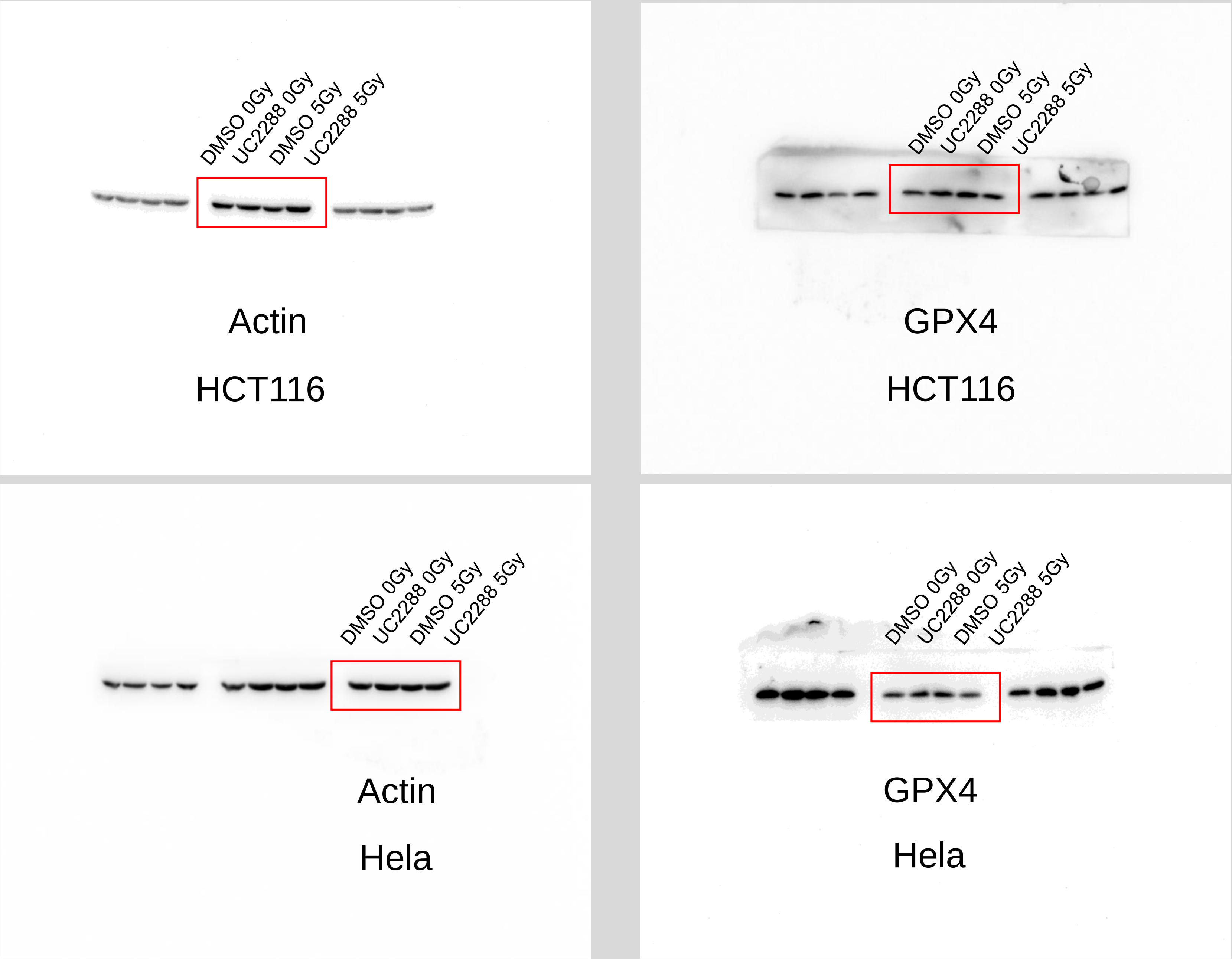
DMSO 0Gy
UC2288 0Gy
DMSO 5Gy
UC2288 5Gy
Actin
HCT116
DMSO 0Gy
UC2288 0Gy
DMSO 5Gy
UC2288 5Gy
GPX4
HCT116
DMSO 0Gy
UC2288 0Gy
DMSO 5Gy
UC2288 5Gy
Actin
Hela
DMSO 0Gy
UC2288 0Gy
DMSO 5Gy
UC2288 5Gy
GPX4
Hela

Figure 4H

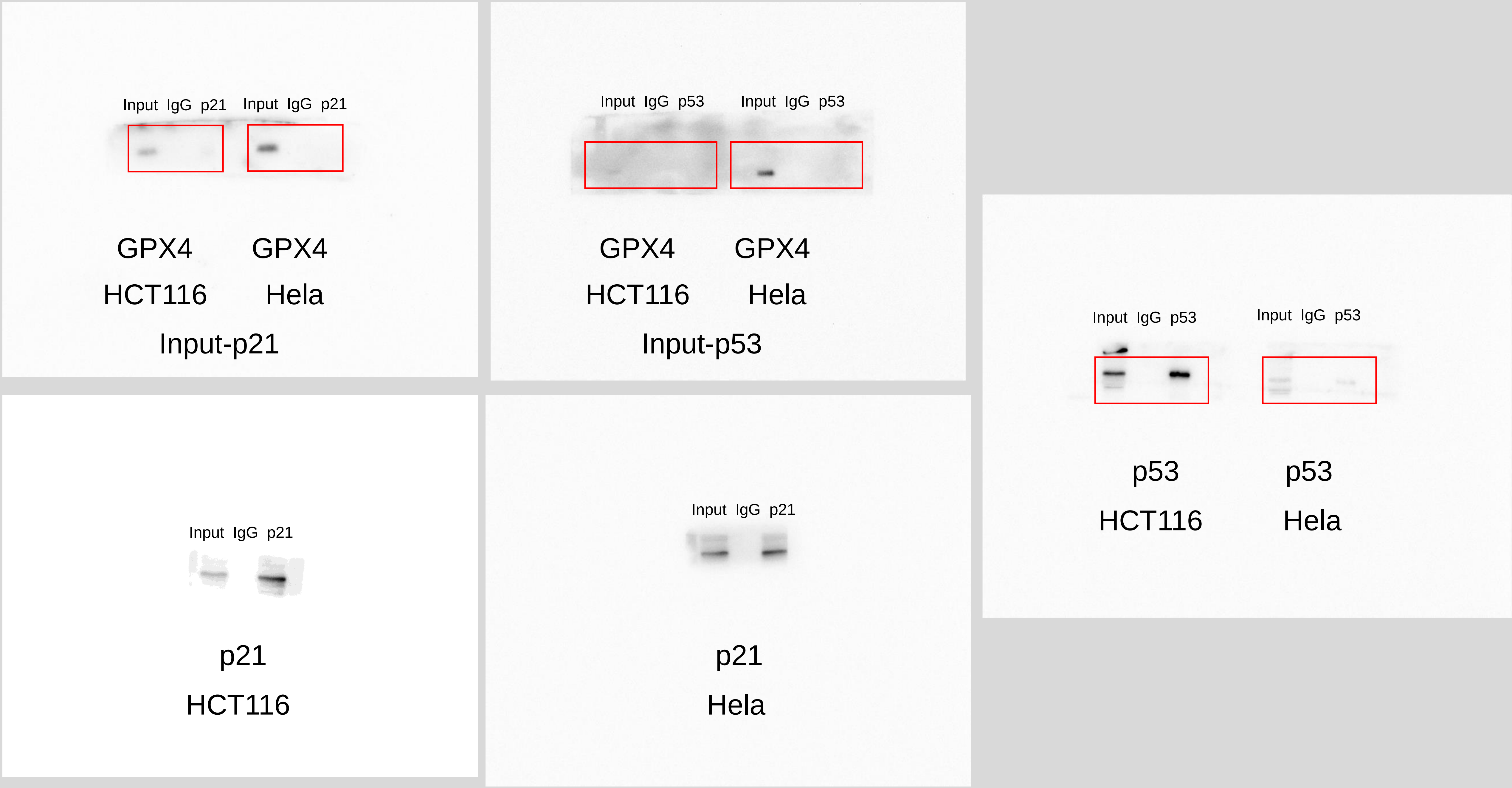
Input IgG p21 Input IgG p21
GPX4 GPX4
HCT116 Hela
Input-p21
Input IgG p53 Input IgG p53
GPX4 GPX4
HCT116 Hela
Input-p53
Input IgG p53 Input IgG p53
p53 p53
HCT116 Hela
Input IgG p21
p21
HCT116
Input IgG p21
p21
Hela

Figure 5E

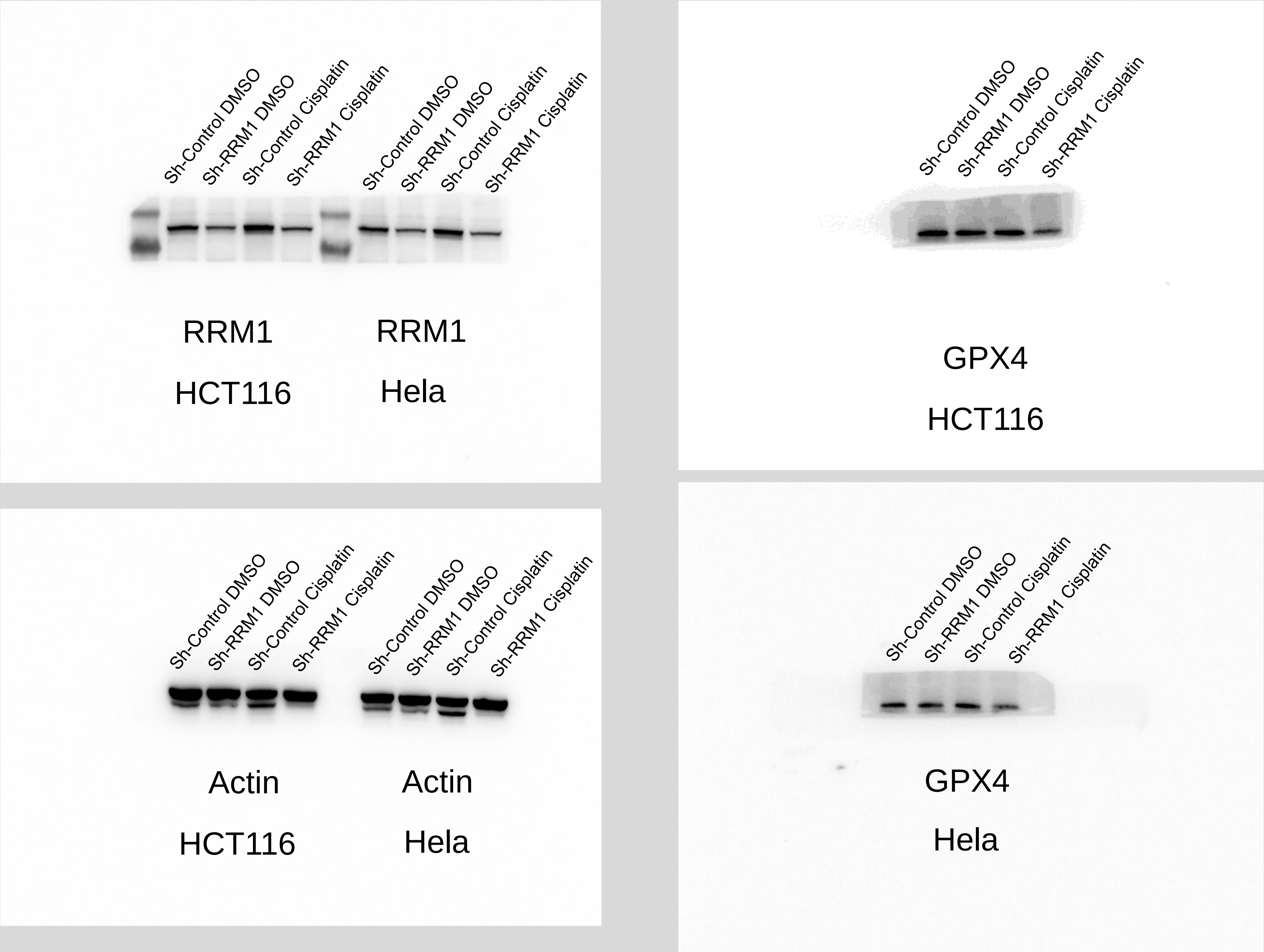
Sh-Control DMSO
Sh-RRM1 DMSO
Sh-Control Cisplatin
Sh-RRM1 Cisplatin
Sh-Control DMSO
Sh-RRM1 DMSO
Sh-Control Cisplatin
Sh-RRM1 Cisplatin
RRM1
RRM1
HCT116
Hela
Sh-Control DMSO
Sh-RRM1 DMSO
Sh-Control Cisplatin
Sh-RRM1 Cisplatin
GPX4
HCT116
Sh-Control DMSO
Sh-RRM1 DMSO
Sh-Control Cisplatin
Sh-RRM1 Cisplatin
Sh-Control DMSO
Sh-RRM1 DMSO
Sh-Control Cisplatin
Sh-RRM1 Cisplatin
Actin
Actin
HCT116
Hela
Sh-Control DMSO
Sh-RRM1 DMSO
Sh-Control Cisplatin
Sh-RRM1 Cisplatin
GPX4
Hela

Figure 6C

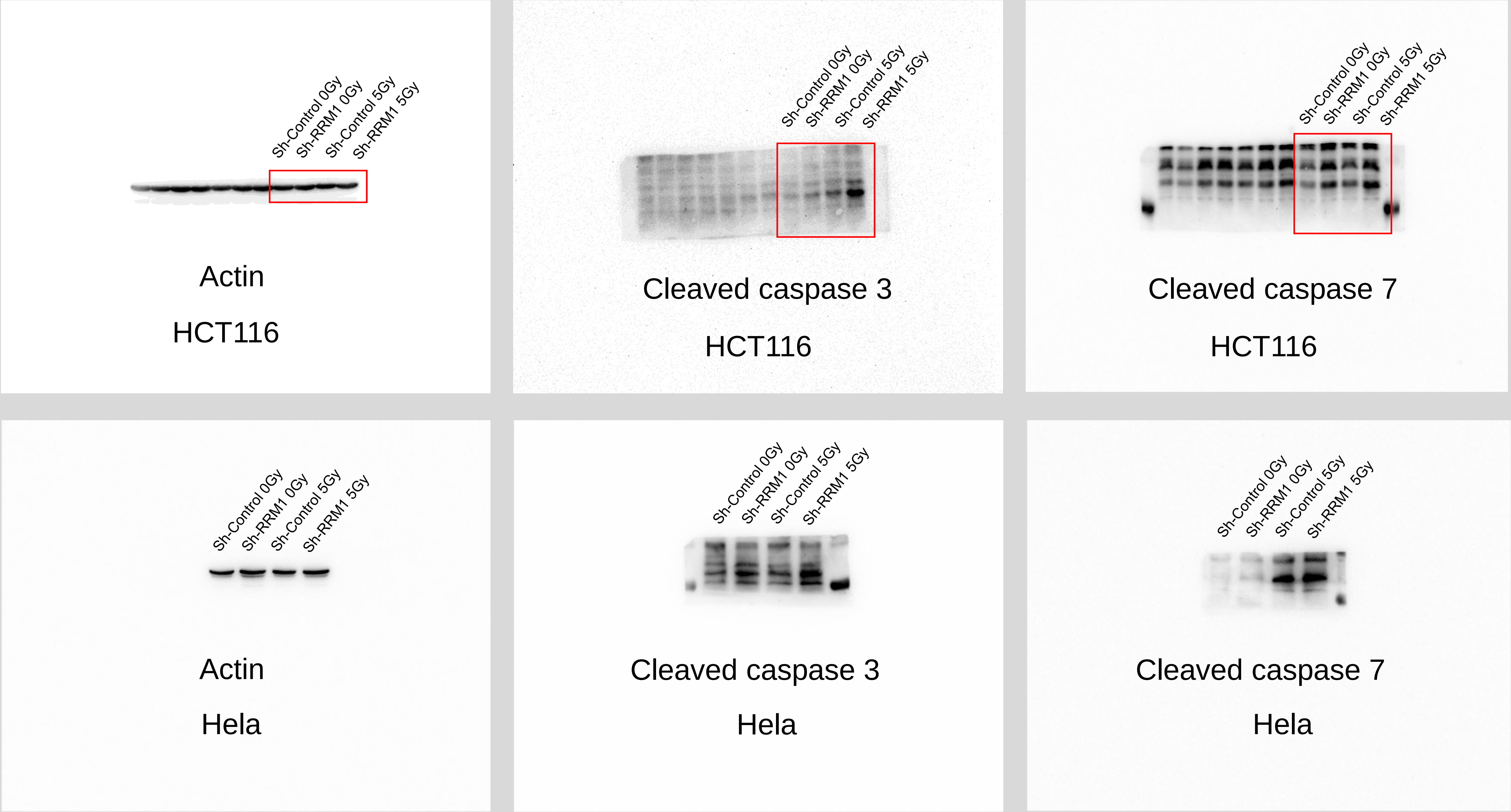

Sh-Control 0Gy
Sh-RRM1 0Gy
Sh-Control 5Gy
Sh-RRM1 5Gy
Actin
HCT116
Cleaved caspase 3
HCT116
Cleaved caspase 7
HCT116
Actin
Hela
Cleaved caspase 3
Hela
Cleaved caspase 7
Hela

Figure 6C

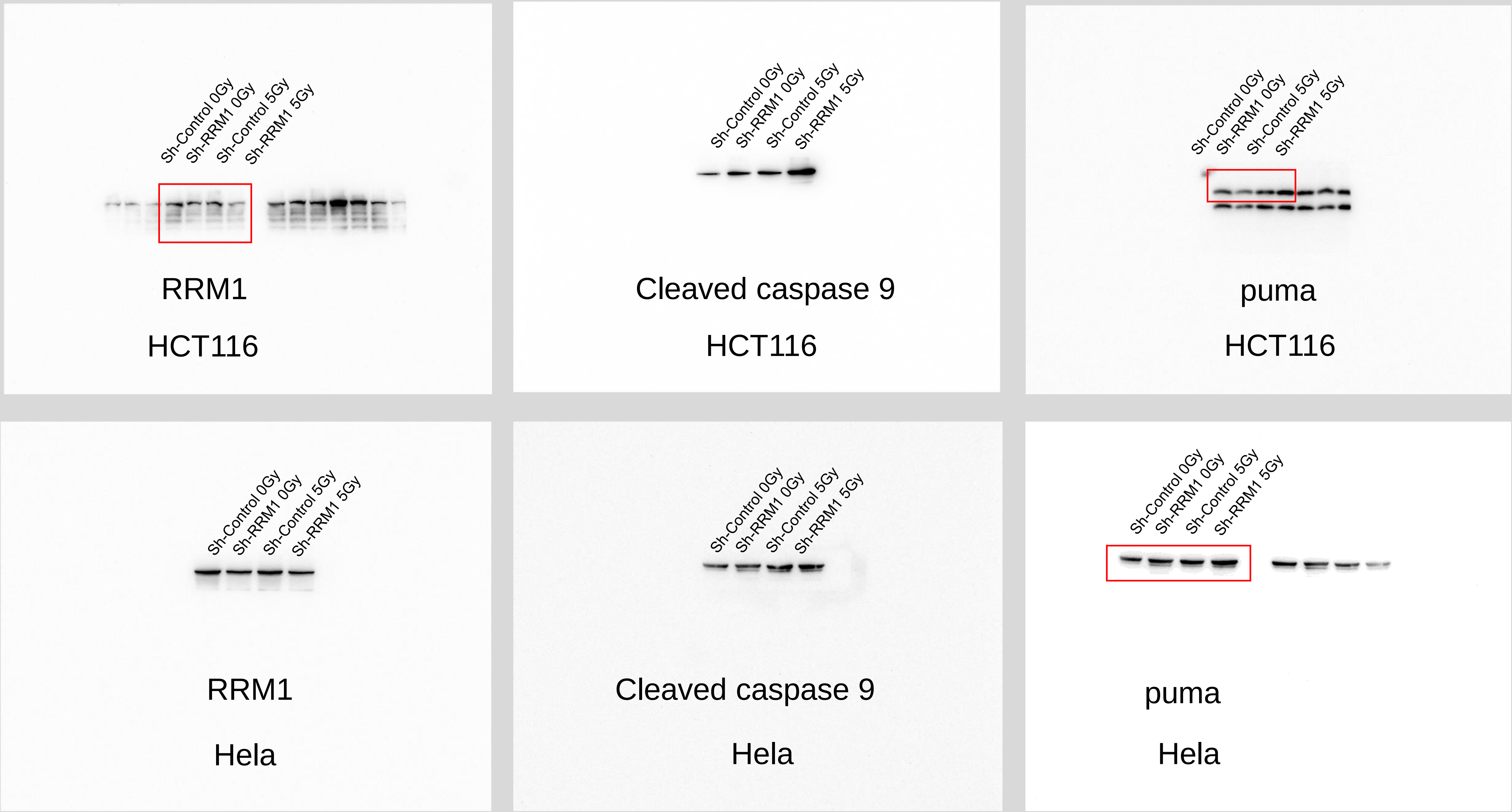
Sh-Control 0Gy
Sh-RRM1 0Gy
Sh-Control 5Gy
Sh-RRM1 5Gy
RRM1
HCT116
Sh-Control 0Gy
Sh-RRM1 0Gy
Sh-Control 5Gy
Sh-RRM1 5Gy
Cleaved caspase 9
HCT116
Sh-Control 0Gy
Sh-RRM1 0Gy
Sh-Control 5Gy
Sh-RRM1 5Gy
puma
HCT116
Sh-Control 0Gy
Sh-RRM1 0Gy
Sh-Control 5Gy
Sh-RRM1 5Gy
RRM1
Hela
Sh-Control 0Gy
Sh-RRM1 0Gy
Sh-Control 5Gy
Sh-RRM1 5Gy
Cleaved caspase 9
Hela
Sh-Control 0Gy
Sh-RRM1 0Gy
Sh-Control 5Gy
Sh-RRM1 5Gy
puma
Hela

Figure 6D

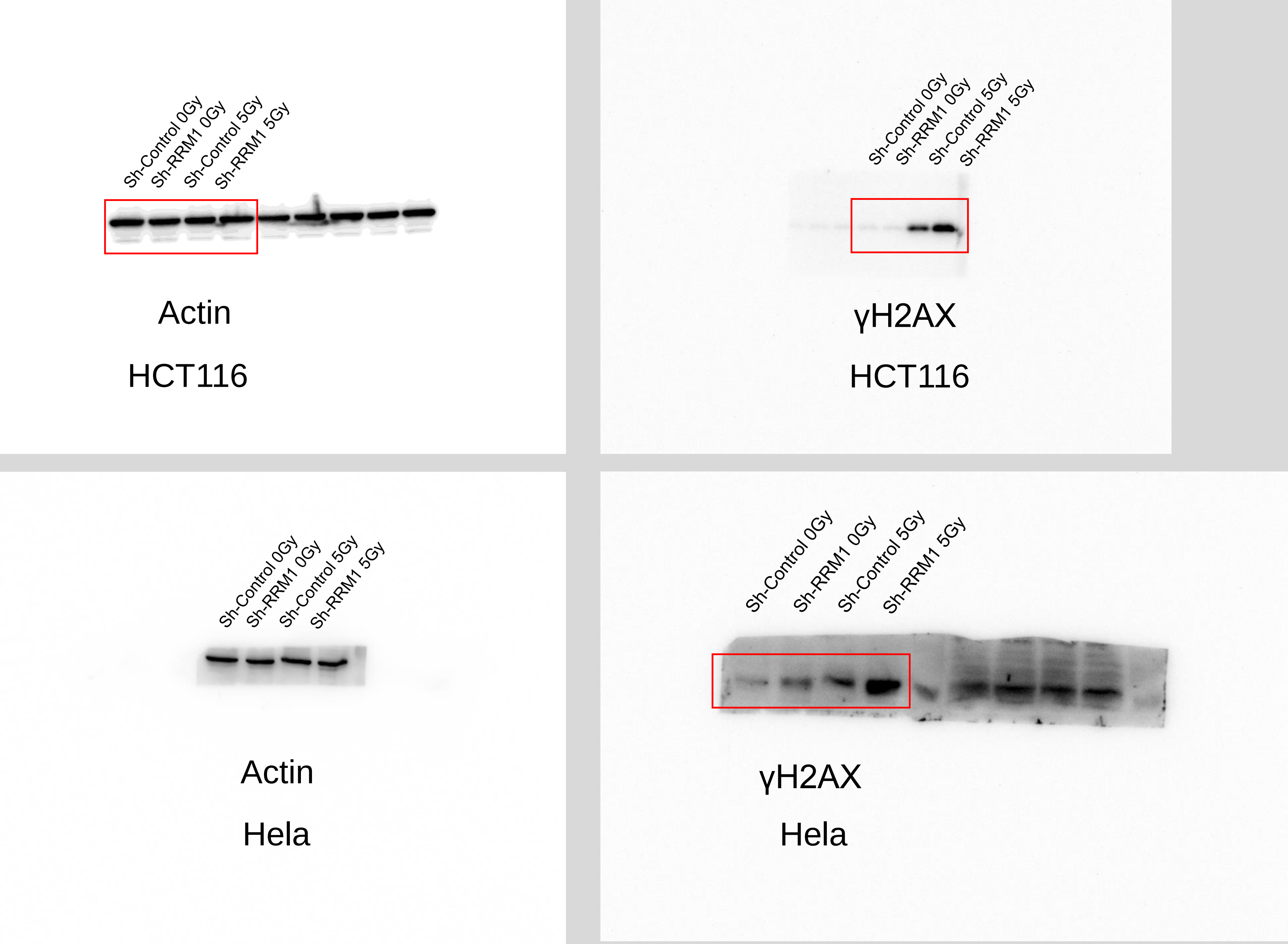
Sh-Control 0Gy
Sh-RRM1 0Gy
Sh-Control 5Gy
Sh-RRM1 5Gy
Actin
HCT116
Sh-Control 0Gy
Sh-RRM1 0Gy
Sh-Control 5Gy
Sh-RRM1 5Gy
γH2AX
HCT116
Sh-Control 0Gy
Sh-RRM1 0Gy
Sh-Control 5Gy
Sh-RRM1 5Gy
Actin
Hela
Sh-Control 0Gy
Sh-RRM1 0Gy
Sh-Control 5Gy
Sh-RRM1 5Gy
γH2AX
Hela

Figure 6F

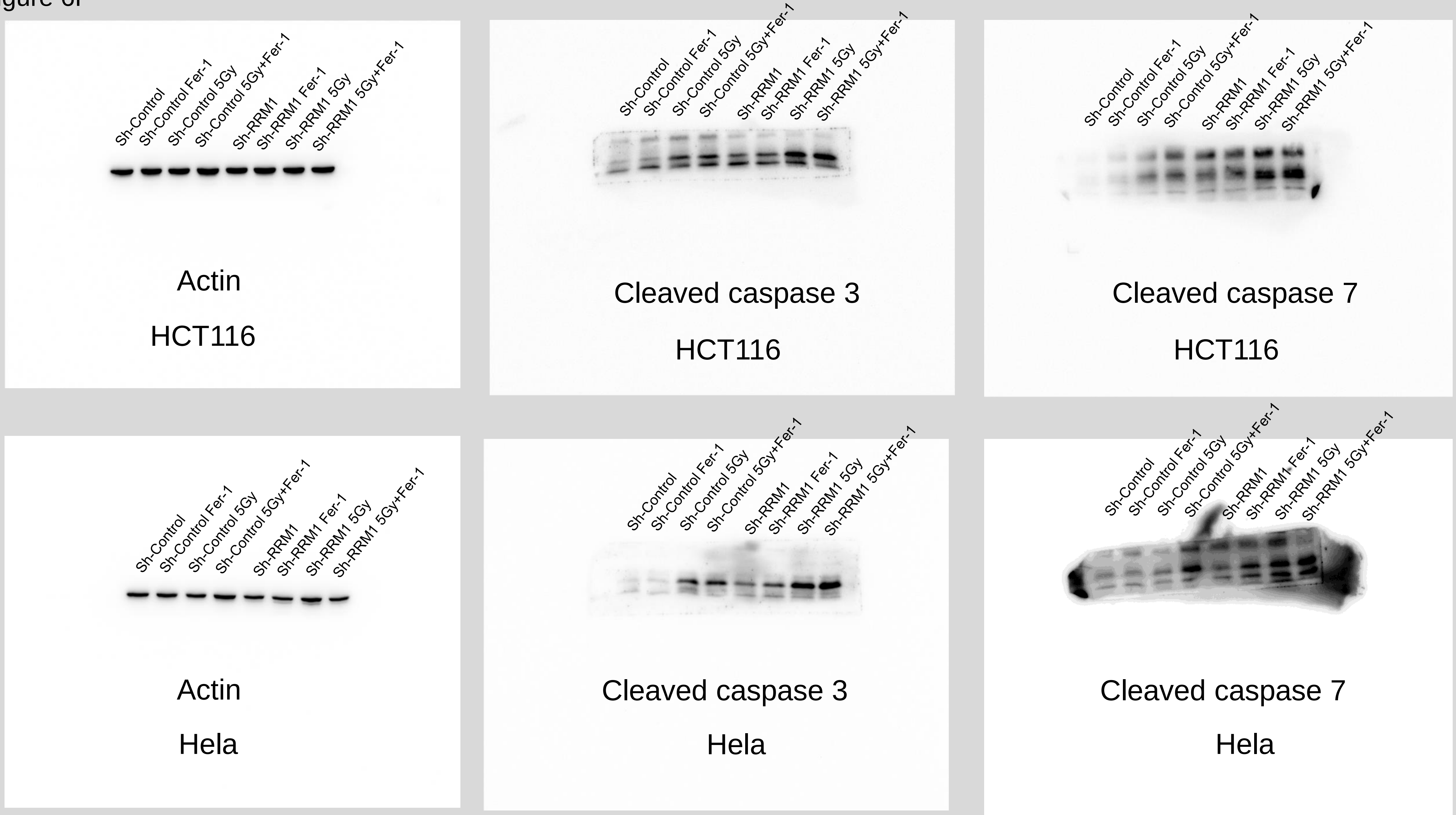

Sh-Control
Sh-Control Fer-1
Sh-Control 5Gy
Sh-Control 5Gy+Fer-1
Sh-RRM1
Sh-RRM1 Fer-1
Sh-RRM1 5Gy
Sh-RRM1 5Gy+Fer-1
Actin
HCT116
Sh-Control
Sh-Control Fer-1
Sh-Control 5Gy
Sh-Control 5Gy+Fer-1
Sh-RRM1
Sh-RRM1 Fer-1
Sh-RRM1 5Gy
Sh-RRM1 5Gy+Fer-1
Cleaved caspase 3
HCT116
Sh-Control
Sh-Control Fer-1
Sh-Control 5Gy
Sh-Control 5Gy+Fer-1
Sh-RRM1
Sh-RRM1 Fer-1
Sh-RRM1 5Gy
Sh-RRM1 5Gy+Fer-1
Cleaved caspase 7
HCT116
Sh-Control
Sh-Control Fer-1
Sh-Control 5Gy
Sh-Control 5Gy+Fer-1
Sh-RRM1
Sh-RRM1 Fer-1
Sh-RRM1 5Gy
Sh-RRM1 5Gy+Fer-1
Actin
Hela
Sh-Control
Sh-Control Fer-1
Sh-Control 5Gy
Sh-Control 5Gy+Fer-1
Sh-RRM1
Sh-RRM1 Fer-1
Sh-RRM1 5Gy
Sh-RRM1 5Gy+Fer-1
Cleaved caspase 3
Hela
Sh-Control
Sh-Control Fer-1
Sh-Control 5Gy
Sh-Control 5Gy+Fer-1
Sh-RRM1
Sh-RRM1 Fer-1
Sh-RRM1 5Gy
Sh-RRM1 5Gy+Fer-1
Cleaved caspase 7
Hela

Figure S1A

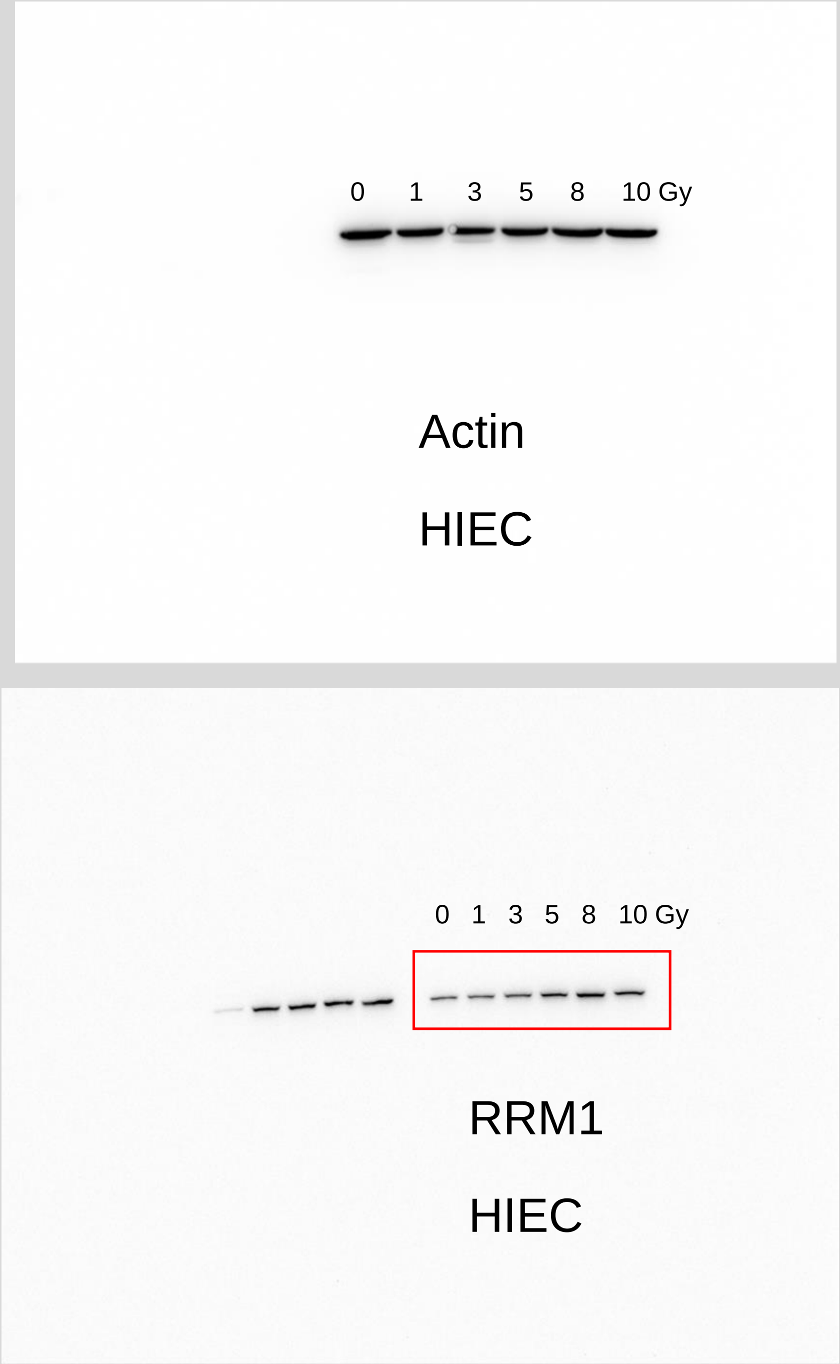

Figure S1B

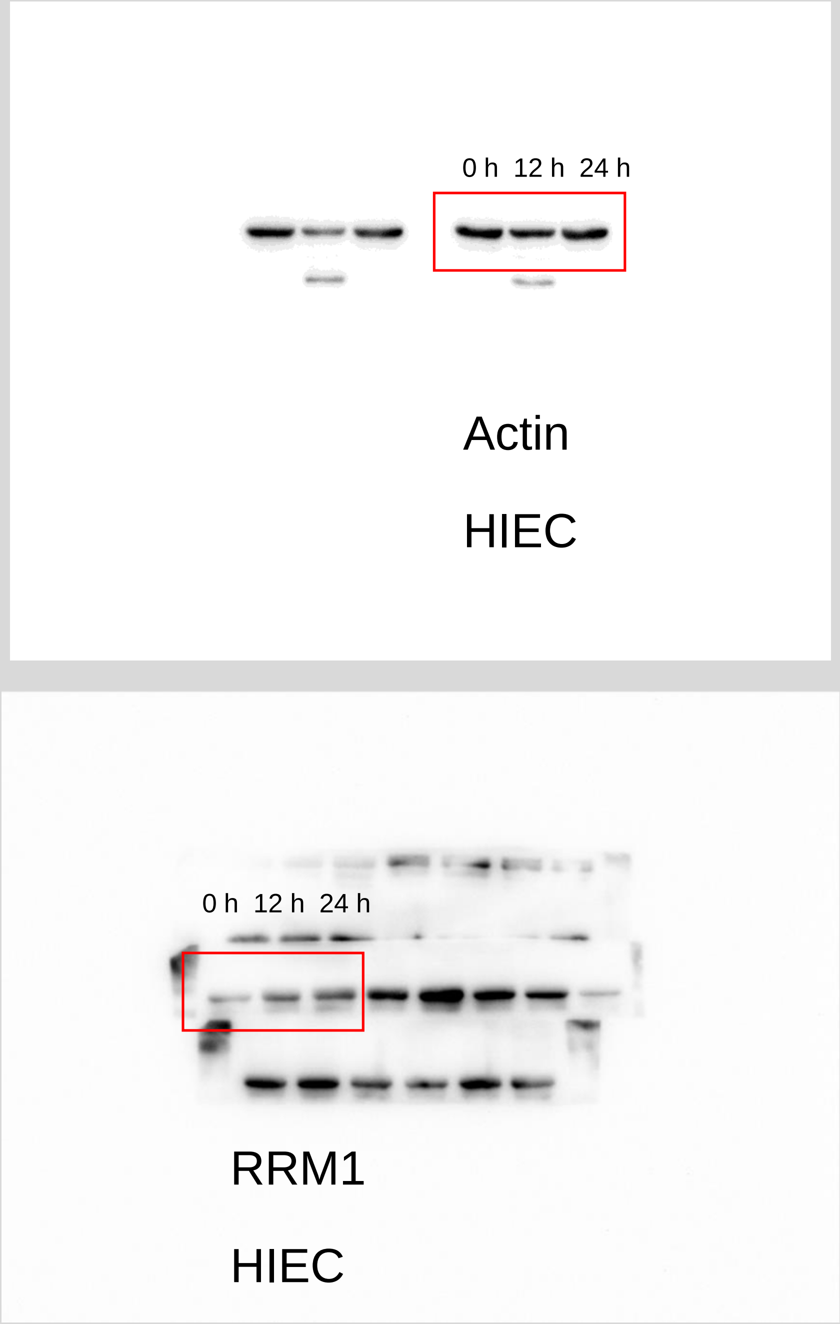

Figure S2A

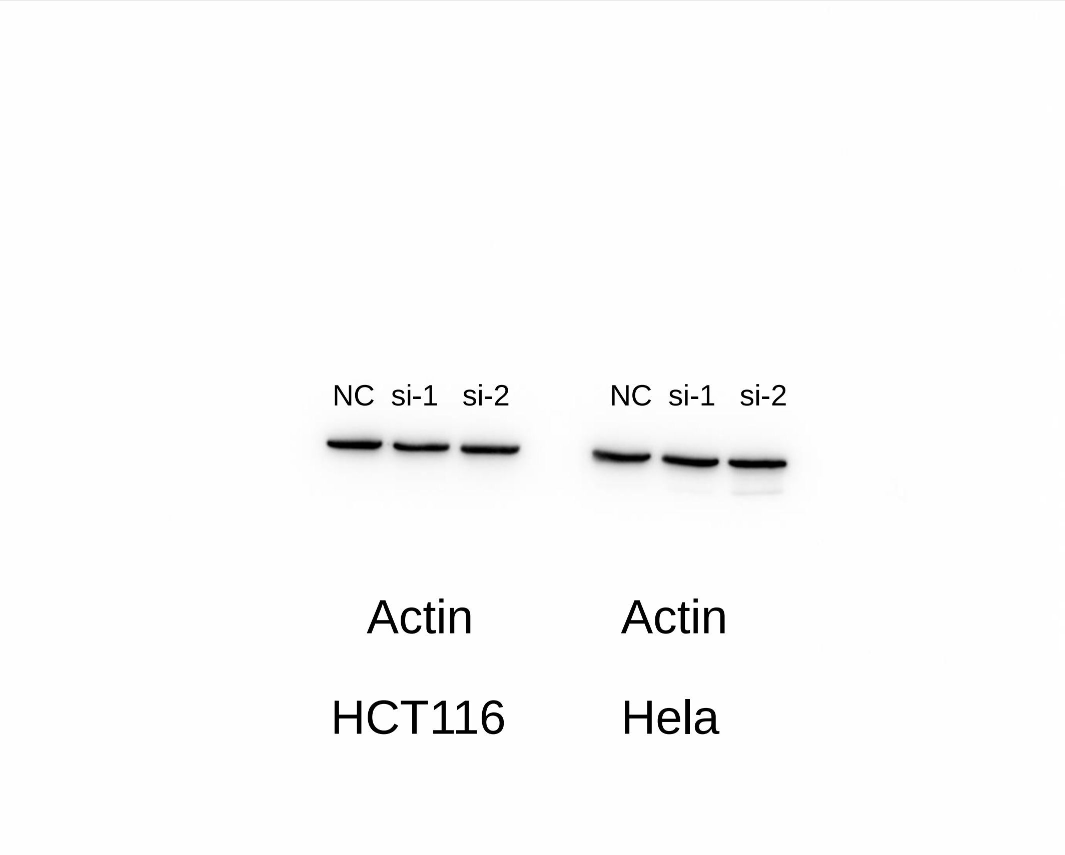
NC si-1 si-2
NC si-1 si-2
Actin
Actin
HCT116
Hela

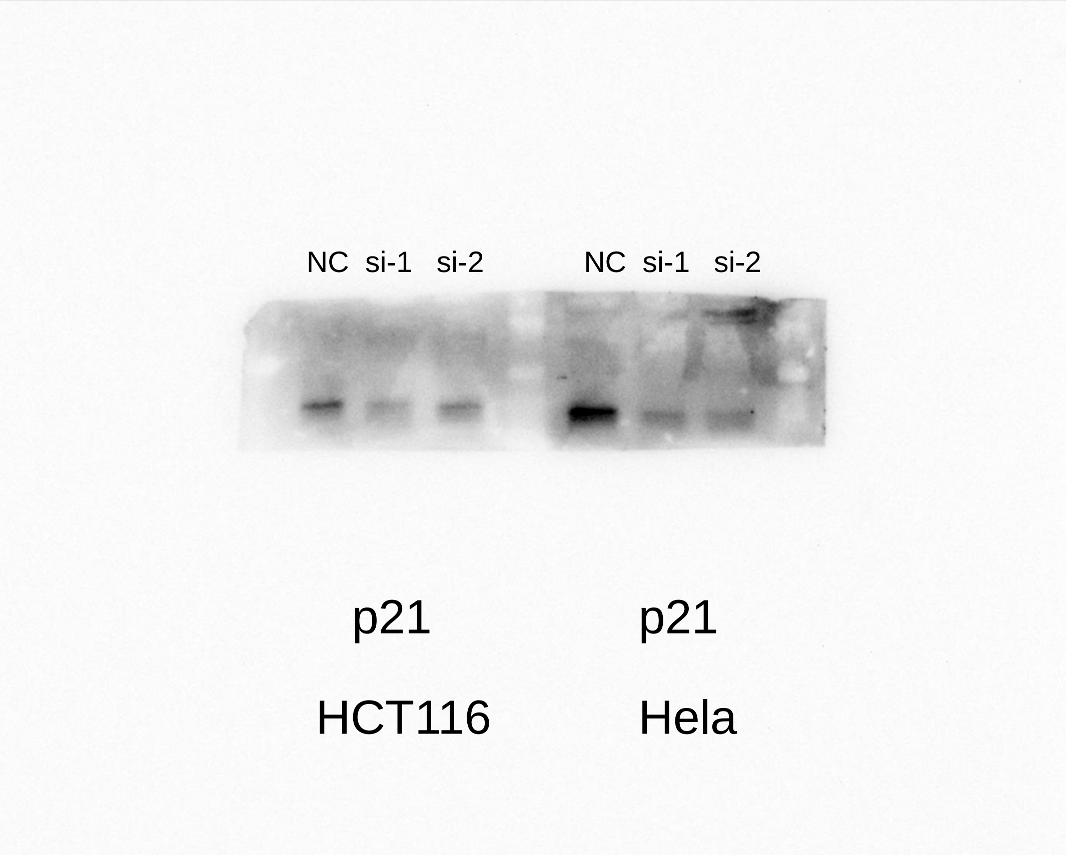
NC si-1 si-2
NC si-1 si-2
p21
p21
HCT116
Hela

Figure S2B

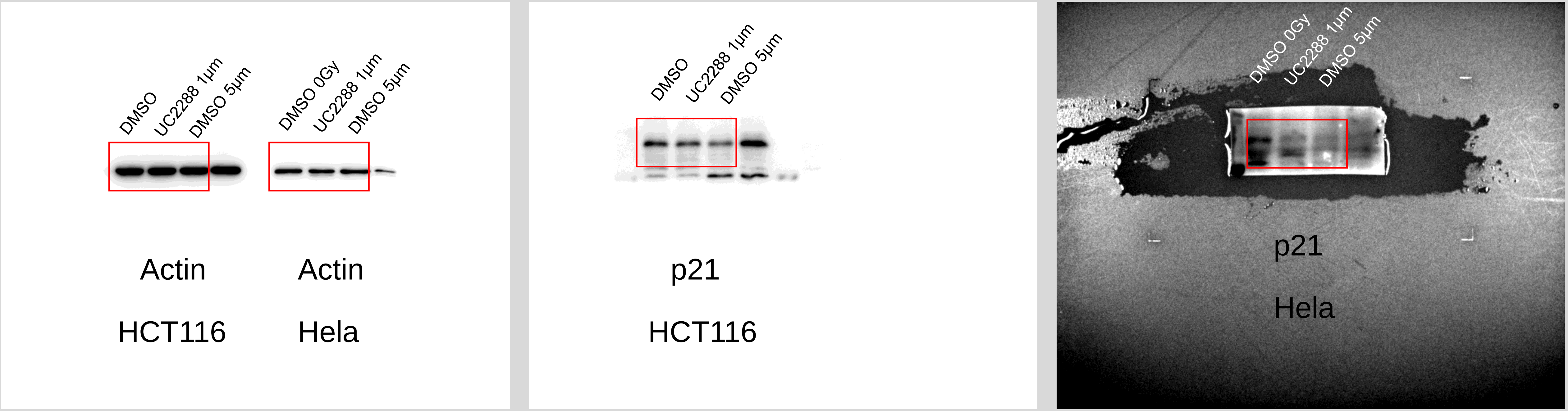
DMSO
UC2288 1μm
DMSO 5μm
DMSO 0Gy
UC2288 1μm
DMSO 5μm
Actin
Actin
HCT116
Hela
DMSO
UC2288 1μm
DMSO 5μm
p21
HCT116
DMSO 0Gy
UC2288 1μm
DMSO 5μm
p21
Hela

Figure S2C

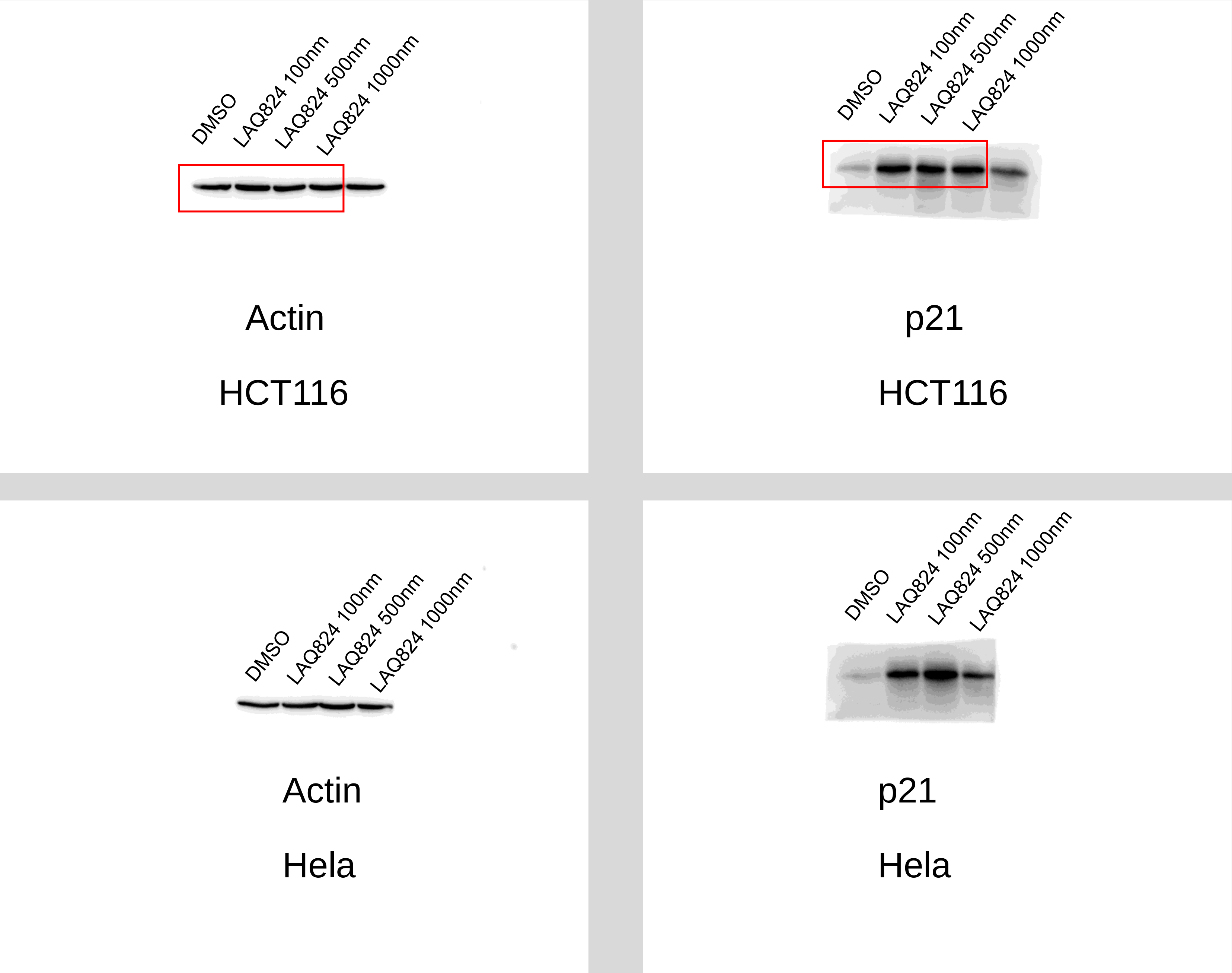
DMSO
LAQ824 100nm
LAQ824 500nm
LAQ824 1000nm
Actin
HCT116
DMSO
LAQ824 100nm
LAQ824 500nm
LAQ824 1000nm
p21
HCT116
DMSO
LAQ824 100nm
LAQ824 500nm
LAQ824 1000nm
Actin
Hela
DMSO
LAQ824 100nm
LAQ824 500nm
LAQ824 1000nm
p21
Hela

Figure S2D

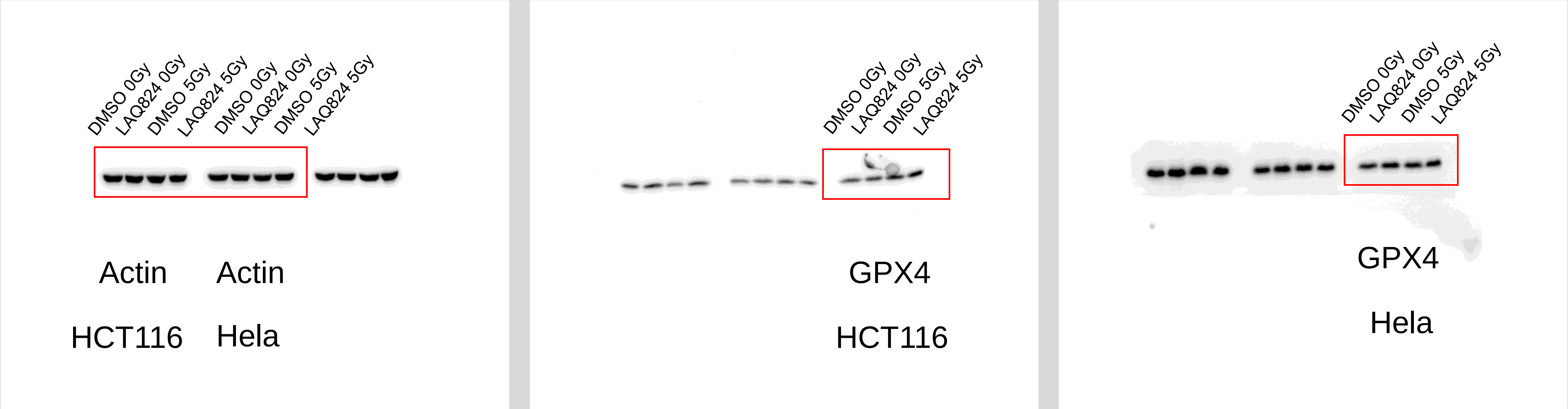
DMSO 0Gy
LAQ824 0Gy
DMSO 5Gy
LAQ824 5Gy
DMSO 0Gy
LAQ824 0Gy
DMSO 5Gy
LAQ824 5Gy
Actin
Actin
HCT116
Hela
DMSO 0Gy
LAQ824 0Gy
DMSO 5Gy
LAQ824 5Gy
GPX4
HCT116
DMSO 0Gy
LAQ824 0Gy
DMSO 5Gy
LAQ824 5Gy
GPX4
Hela

Figure S3C

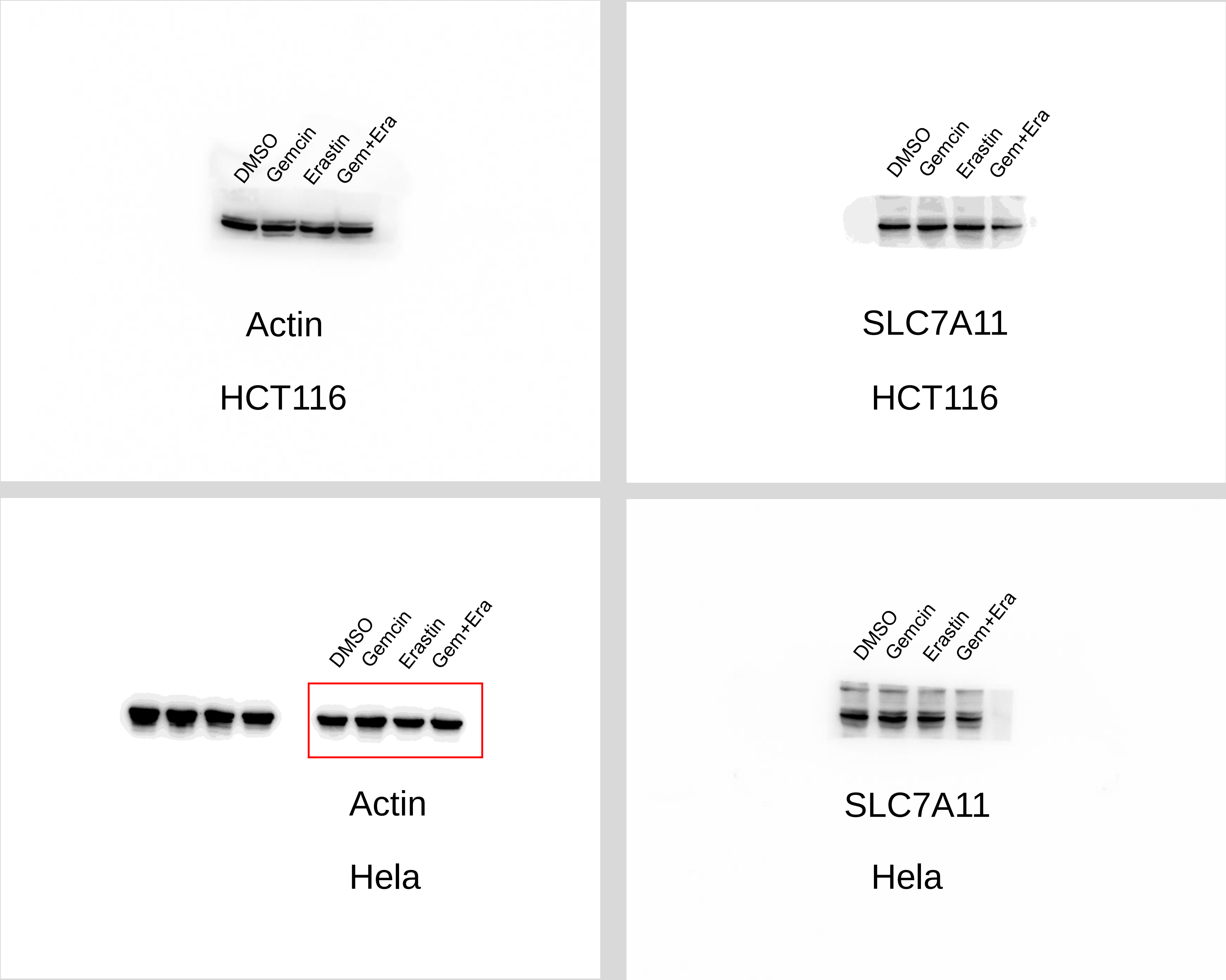
DMSO
Gemcin
Erastin
Gem+Era
Actin
HCT116
DMSO
Gemcin
Erastin
Gem+Era
SLC7A11
HCT116
DMSO
Gemcin
Erastin
Gem+Era
Actin
Hela
DMSO
Gemcin
Erastin
Gem+Era
SLC7A11
Hela
